# Severe congenital myasthenic syndromes caused by agrin mutations affecting secretion by motoneurons

**DOI:** 10.1007/s00401-022-02475-8

**Published:** 2022-08-10

**Authors:** Arnaud Jacquier, Valérie Risson, Thomas Simonet, Florine Roussange, Nicolas Lacoste, Shams Ribault, Julien Carras, Julian Theuriet, Emmanuelle Girard, Isabelle Grosjean, Laure Le Goff, Stephan Kröger, Julia Meltoranta, Stéphanie Bauché, Damien Sternberg, Emmanuel Fournier, Anna Kostera-Pruszczyk, Emily O’Connor, Bruno Eymard, Hanns Lochmüller, Cécile Martinat, Laurent Schaeffer

**Affiliations:** 1 Pathophysiology and Genetics of Neuron and Muscle, Faculté de Médecine Lyon Est, CNRS UMR 5261, INSERM U1315, Université Lyon1, Lyon, France; 2 Hospices Civils de Lyon, Groupement Est, Bron, France; 3 INSERM/UEPS UMR 861, Paris Saclay Université, I-STEM, 91100 Corbeil-Essonnes, France; 4 Service de Médecine Physique et de Réadaptation, Hôpital Henry Gabrielle, Hospices Civils de Lyon, 69230 Saint-Genis-Laval, France; 5 Department of Physiological Genomics, Biomedical Center, Planegg, Martinsried, Germany; 6 Inserm U 1127, CNRS UMR 7225, UPMC Université Paris 06 UMR S 1127, Institut du Cerveau et de la Moelle Épinière, ICM, Sorbonne Universités, 75013 Paris, France; 7 APHP, UF Cardiogénétique et Myogénétique, Service de Biochimie Métabolique, Groupe Hospitalier Pitié-Salpêtrière, Paris, France; 8 AP-HP, Hôpital Pitié-Salpêtrière, 75013 Paris, France; 9 Département d’Éthique de l’Université et des enseignements de Physiologie de la Faculté de Médecine Pitié-Salpêtrière, 75013 Paris, France; 10 Department of Neurology, Medical University of Warsaw, Warsaw, Poland; 11 Division of Neurology, Department of Medicine, Children’s Hospital of Eastern Ontario Research Institute, The Ottawa Hospital and Brain and Mind Research Institute, University of Ottawa, Ottawa, Canada

## Abstract

Congenital myasthenic syndromes (CMS) are predominantly characterized by muscle weakness and fatigability and can be caused by a variety of mutations in genes required for neuromuscular junction formation and maintenance. Among them, *AGRN* encodes agrin, an essential synaptic protein secreted by motoneurons. We have identified severe CMS patients with uncharacterized p.R1671Q, p.R1698P and p.L1664P mutations in the LG2 domain of agrin*.* Overexpression in primary motoneurons cultures in vitro and in chick spinal motoneurons in vivo revealed that the mutations modified agrin trafficking, leading to its accumulation in the soma and/or in the axon. Expression of mutant agrins in cultured cells demonstrated accumulation of agrin in the endoplasmic reticulum associated with induction of unfolded protein response (UPR) and impaired secretion in the culture medium. Interestingly, evaluation of the specific activity of individual agrins on AChR cluster formation indicated that when secreted, mutant agrins retained a normal capacity to trigger the formation of AChR clusters. To confirm agrin accumulation and secretion defect, iPS cells were derived from a patient and differentiated into motoneurons. Patient iPS-derived motoneurons accumulated mutant agrin in the soma and increased XBP1 mRNA splicing, suggesting UPR activation. Moreover, co-cultures of patient iPS-derived motoneurons with myotubes confirmed the deficit in agrin secretion and revealed a reduction in motoneuron survival. Altogether, we report the first mutations in *AGRN* gene that specifically affect agrin secretion by motoneurons. Interestingly, the three patients carrying these mutations were initially suspected of spinal muscular atrophy (SMA). Therefore, in the presence of patients with a clinical presentation of SMA but without mutation in the *SMN1* gene, it can be worth to look for mutations in *AGRN*.

**Supplementary Information:**

The online version contains supplementary material available at 10.1007/s00401-022-02475-8.

## Introduction

The neuromuscular junction (NMJ) is a specialized synapse with a complex molecular architecture that ensures reliable conversion of the nerve action potential into muscle contraction. Genetic alterations of neurotransmission resulting from presynaptic, synaptic, or post-synaptic defects cause congenital myasthenic syndromes (CMS [MIM 608931]), a heterogeneous family of neuromuscular disorders characterized by skeletal muscle weakness and fatigue [13, 41]. The onset of CMS is typically early in life, and ophthalmoparesis, ptosis, and bulbar weakness are frequently observed. CMS patients are usually devoid of neurogenic signs and can often improve with drugs targeting the neuromuscular junction (NMJ) such as cholinesterase inhibitors or β2-sympathomimetics.

Diagnosis of CMS is challenging and relies on clinical evaluation, morphological studies of muscle and peripheral nerves, electromyography showing decrement on repetitive muscle stimulation or abnormal jitter and blocking on single-fiber electromyography. Moreover, the absence of serum antibodies against muscle acetylcholine receptors (AChR) or tyrosine kinase receptor MUSK, the demonstration of AChR deficiency, and, more recently, findings from molecular genetic studies are arguments in favor of CMS. Indeed, mutations in a panel of genes coding for pre- and post-synaptic proteins, as well as proteins located in the synaptic cleft, have been shown to cause CMS [13, 41]. Since 2009, several mutations causing CMS have been identified in the gene coding for agrin [18, 23, 27, 33, 35, 45, 47, 50].

Agrin is a very large heparan-sulfate proteoglycan that occurs in multiple isoforms generated by alternative splicing, with diverse functions in different tissues [36]. In motoneurons (MNs), specific alternative splicing at the A/y and B/z sites confers agrin the ability to induce AChR clustering: inclusion of exon 32 at the A/y site causes the insertion of 4 amino acids in the LG2 domain, and inclusion of exon 36 at the B/z site inserts 8 amino acids in the LG3 domain.

Neural agrin expressed by MNs is secreted into the synaptic basal lamina of the NMJ where it controls the organization of the post-synaptic domain by binding to the low-density lipoprotein receptor-related protein 4 (LRP4), which thereby activates the tyrosine kinase receptor MuSK [15, 24, 49]. Once activated, MuSK recruits a plethora of signaling molecules that orchestrate post-synaptic organization, including AChR aggregation and AChR subunit genes’ expression [46].

Since 2009, several mutations in *AGRN* have been reported to cause CMS. Most of them correspond to missense mutations, homoallelic or compound heteroallelic, and are located in the LG2 domain, but mutations were also found in the N-terminal laminin binding, SEA, LG1 and LG3 domains [18, 23, 27, 33, 35, 45, 47, 50]. Clinical manisfestations and symptoms severity are very variable among patients. The age of onset ranges from birth to the third decade. The first symptoms reported by the patients were highly variable, comprising limb-girdle, proximo-distal weakness or difficulty to run, distal weakness with distal amyotrophy, fatigue, dropped head syndrome, ptosis, apneic episodes and stridor. The spectrum of motor impairment is also variable, ranging from no acquisition or loss of independent ambulation to difficulties in running without evident weakness at neurological examination. Ptosis is frequently but not systematically present. Limitation of external oculomotricity can be present, but no abnormal pupillary response was reported. Some patient have normal respiratory function, while others present defects ranging from exercise-induced breathlessness to respiratory failure requiring tracheostomy. Bulbar involvement with weakness in chewing or swallowing is not systematically present. Two patients with onset at birth or during the neonatal period were reported to show intellectual impairment [35, 45]. When evaluated, repetitive nerve stimulation showed that significative decrement is all patients. For some patients, an increment post-exercise or under high-frequency stimulation was noted [33]. Needle electromyography predominantly showed myopathic changes, but neurogenic changes were reported in one patient associated with decreased conduction velocities [45], and spontaneous activities including positive sharp waves and fibrillations were reported in some patients [33, 45, 50].

Until now, the pathogenicity of agrin mutations was mainly analyzed regarding their effect on AChR clustering in cultured myotubes, MuSK phosphorylation and Heparin binding. In 2019, the V1727F mutation in the LG2 domain was investigated more in depth and showed reduced secretion as well as reduced binding to heparin and LRP4. In silico modeling predicted that the mutation disrupted the A/y slice insert, thus explaining the reduced affinity for heparin and LRP4 [42].

Possible effects of *AGRN* mutations in MNs have not been reported yet. Here, we have identified three CMS patients showing unusual signs of severe and chronic denervation at the EMG. Sequencing candidate genes revealed two heteroallelic missense mutations in the *AGRN* gene in the first family (Patient 1: mutation L1, p.R1671Q, mutation L2, p.R1698P) and a homoallelic mutation in the second family (Patient 2.1 and 2.2: mutation LM, p.L1664P). All mutations were located in the LG2 domain. Molecular characterization of the mutant agrins revealed that they all affected agrin secretion by neurons. This finding provides the first evidence of CMS caused by *AGRN* mutations causing retention in MNs and neurotoxicity.

## Materials and methods

### Patient and electrophysiological features

Clinical evaluation, muscle biopsies, and blood samples were obtained after informed written consent and were secured following the protocol approved by national ethic committees (DC-2019-3835 and AC-2021-4496). Samples collection was realized under national and European regulations (Article L1243-3 and L1243-4). Electromyography (EMG), including repetitive nerve stimulation (RNS), nerve conduction studies, and electromyography, were carried out in selected muscles using standardized protocols [1]. Compound muscle action potentials were recorded at rest and after exercise. A pathological decrement was considered when the amplitude and surface were decreased by 5%, and an increment when they were increased by 20%.

### Patient muscle specimens

Deltoid muscle specimens from Patient 1 were obtained by open biopsy by determining the NMJ-rich zone by the small twitch provoked by the scalpel’s tip on the muscle fascicles’ surface. Using the classical Koelle method, the presence of NMJs was confirmed on a longitudinal strip of the biopsy by revealing cholinesterase activity [7], from origin to insertion, from patients and control subjects without muscle disease who were undergoing thoracic surgery. Whole mounts of specimens fixed with 4% paraformaldehyde (PFA) in phosphate-buffered saline (PBS; #D8537, Sigma) were stained for AChR and for neurofilaments and were observed using the laser-scanning microscope (LSM) 510 confocal laser-scanning microscope equipped with a 63 × objective (LSM 510, Carl Zeiss, Inc) after mounted in Vectashield® (H-1000, Vector Laboratories). In addition, to analyze the muscle-fiber morphology, transversal cryosections of 10 μm were stained with conventional hematoxylin–eosin.

### Mutations analysis and sequencing procedures

Genomic DNA was isolated using a blood DNA extraction kit according to the manufacturer's recommendations (Promega, Mannheim, Germany). The 36 exons of *AGRN* and their flanking intronic regions were amplified by PCR and sequenced. PCR-amplified fragments were purified, fluorescently labeled with dideoxy terminators (BigDye Terminator v3.1 Cycle Sequencing Kit, Applied Biosystems), and run on an Applied Biosystems model 3730XL DNA Analyser. The GenBank reference number used for comparison of the mRNA sequence of the *AGRN* exons is NM_198576.3. Any variant was confirmed as a disease-causing mutation by analysis of segregation in affected family members, conservation of the residue among species and isoforms, and absence in at least 200 control chromosomes of healthy adults. Extensive sequencing for *AGRN* revealed frequent variants that were thus considered to be polymorphisms.

### Plasmid constructs and mutagenesis

We generated a human mini-agrin construct, derived from chick mini-agrin which is known to aggregate AChR [31]. It is comprised of the secretion signal peptide, the NTA domain and the first follistatin/kazak-like domain, as well as the whole C-terminal part of the protein, from the first EGF-like domain to the end. This gave rise to a 125 kD protein instead of the 220 kD full-length. This corresponds to nucleotides 51–797 and 4035–6185 of the cDNA NM_198576.3, plus the (*y*) and (*z*) insertions. The nucleotide sequence was changed to optimized protein production in HEK293, without changing the amino acid sequence, by GenScript corp. (120 Centennial Ave.Piscataway, NJ 08854, USA) (sequence on request). It also introduced the patient mutations p.R1671Q (L1), p.R1698P (L2) or p.L1664P (LM) into the mini-agrin cDNA. The Full-length (FL) human wild-type agrin cDNA was kindly provided by Dr. Angela Vincent (Oxford, England) and the mutations were introduced by GenScript corp. For the expression studies in primary neurons or chick embryos, the mini or the FL-agrin cDNA was subcloned into the pCAGIG vector gifted from Connie Cepko (Addgene plasmid # 11159) [28]. This plasmid pCAGIG is a bicistronic plasmid containing an internal ribosomal entry site that allows the simultaneous expression of human mini-agrin and eGFP and, thus, the identification of transfected cells.

### Cell cultures

In basal conditions, HEK293 and neuroblastoma SHEP cells (RRID:CVCL_0524; an epithelioid subclone of the human neuroblastoma line SK-N-SH) [40] were all cultured in Dulbecco's Modified Eagle’s Medium (DMEM) supplemented with 10% fetal bovine serum (FBS, Thermo Fisher Scientific) and 100 U/mL penicillin/streptomycin (Thermo Fisher Scientific). Transient transfection was achieved with Jetprime® reagent (Polyplus Transfection) following the manufacturer’s conditions. For culturing, freezing, and thawing of the mouse myoblasts C2C12 (ATCC CRL-1772), we follow the same instructions as described in Pęziński et al., 2020 [38]. Cell cultures are regularly checked for the absence of mycoplasma contamination by commercially available mycoplasma detection tests (MycoAlert Plus, # LT07-705, Lonza or PlasmoTest, # rep-pt1, Invivogen) according to the manufacturer’s instructions.

### Fractionation by solubility

To assess the presence of insoluble material, proteins were first extracted in 1% Triton X-100 and 0.25% deoxycholate (50 mM Tris pH8 150 mM NaCl 10 mM EDTA 1% Triton X-100 and 0.25% deoxycholate) for 30 min at 4 °C and centrifuged at 10000 g for 10 min. The supernatant was collected as the Triton soluble fraction. The pellet was resuspended in 2% SDS (i.e., the Laemmli sample buffer, 62.5 mM Tris pH6.5, 10 mM EDTA, 10% glycerol, 2% SDS, 100 mM DTT), and heated at 95 °C for 15 min. After 20 min centrifugation at 20000 *g*, the supernatant was kept as the SDS soluble fraction. Finally, the pellet was resuspended in the same SDS buffer and sonicated for 1 min before loading on the gel. This constituted the SDS insoluble fraction. For each fraction in each condition, we loaded the same proportion of the sample.

### Co-immunoprecipitation

Forthy-eight hours after transfection, cells were harvested, resuspended in 300 µL lysis buffer (50 mM Tris pH8, 150 mM NaCl, 2 mM EDTA, 1% Triton X-100, 0.25% DOC, Roche proteases inhibitors cocktail) per 10 cm plates, and rotated on a wheel at 4 °C during 30 min. After 10 min centrifugation at 20000 *g*, 1 mg of supernatant was incubated overnight at 4 °C with primary antibody (5 µl rabbit polyclonal anti-GRP78 Proteintech 11587–1-AP) for immunoprecipitation. Then, 20 µL beads protein A (Protein A-Sepharose® 4B, Sigma P9424) were incubated 3 h before being washed three times in lysis buffer. Immunoprecipitated proteins were eluted in Laemmli Sample buffer and resolved by SDS-PAGE.

### Primary motoneuron culture

Spinal MNs were prepared from E12.5 OF1 mice embryos as described by Henderson et al. [17] with some modifications [19–21]. Briefly, the anterior horns of the embryo were dissected in HBSS (Thermo Fisher Scientific) supplemented by 4.5 g/L glucose and 7 mM HEPES (Invitrogen). Motoneurons were purified using a 6% OptiPrep density gradient medium (#D1556, Sigma). Then, MNs were resuspended in supplemented Neurobasal medium (Invitrogen) containing 1 ng/mL brain-derived neurotrophic factor (#450–02, Peprotech), 1 ng/mL glial cell line-derived neurotrophic factor (#450–51, Peprotech), and 10 ng/mL ciliary neurotrophic factor (#450–50, Peprotech), and were seeded on polyornithine/laminin-coated glass coverslips (#P8638 and #L2020, Sigma). After 2 days in vitro, plasmid transfections were done by magnetofection following the manufacturer’s recommendations (OZBiosciences). Two or four days later, MNs were fixed using 4% PFA for 20 min and washed with PBS.

### *In ovo* electroporation and histological analyses on chick embryos

*In ovo* electroporation of chick embryos (*Gallus*; EARL Morizeau, Dangers, France) was performed as previously described [21]. Briefly, the constructs were introduced into the lumen of the neural tube at the caudal level in stage HH14-15 embryos [16]. Stage HH24-26 chick embryos were harvested and isolated in sterile PBS and defined fragments of the neural folds where the eGFP expression was checked, removed, fixed in 4% PFA, and embedded in 7.5% gelatin/15% sucrose. For immunostaining, cryosections (20 μm) were incubated with primary antibody diluted in 3% bovine serum albumin (BSA) blocking solution (in PBS/0.25% Triton X-100) at 4 °C overnight and reveled with the appropriate secondary antibody incubated in blocking solution for 2 h at room temperature.

### Preparation of whole-cell extracts and conditioned media for in vitro acetylcholine receptor clustering assay

To analyze the secretion of wild-type or mutant FL-agrin, the pCAGIG vectors were similarly transfected into HEK293 cells cultured into a 6-well plate. Four hours post-transfection, cells were cultured into a minimum volume (800 µL) of DMEM medium supplemented with 2% horse serum (HS) and 3.7 mg/mL Glucose (#A2494001, Thermo Fisher Scientific). Forty-eight hours post-transfection, the similarity of the cell transfection rate between the different conditions was verified by the GFP fluorescence rate. The conditioned media were sampled, and the cells were delicately washed with PBS. The whole-cell extract (WCE) was then taken in 800 µL of PBS using a cell scraper before being submitted to the bioruptor with the following conditions (15 cycles of 30 s at high intensity).

### Agrin protein quantification

To quantify agrin in conditioned media or in WCE, protein extracts were analyzed by dot-blot (Bio Dot Apparatus, Bio-Rad) in triplicates on 0.45 µm pore nitrocellulose membranes. Mini-agrin proteins were immunostained with the anti-human agrin antibody (1:10.000) in PBS with 5% BSA overnight after 1 h blocking (5% milk in PBS) and revealed by fluorescent secondary antibody (StarBright, Bio-Rad) on a ChemiDoc imager (Bio-Rad). Full-length agrin fused with eGFP was directly detected on a ChemiDoc with a 488 nm filter. Quantification of the dot was done by Image Lab 6 software (Bio-Rad). Secretion rate of agrin was calculated as the amount of agrin in conditioned media divided by the total amount of agrin (i.e., the sum of the amount of agrin in conditioned media and in WCE).

### Acetylcholine receptor clustering assay

For myoblast fusion, we use 4-well chamber slides (LabTek #177437, Thermo Fisher Scientific) coated with 0.1% gelatin. 8.5 × 10^5^ C2C12 myoblasts were seeded per well (cell density 500,000 cells/cm^2^) and myoblast differentiation into myotubes was induced 24 h after cell seeding by gentle replacement of the culture medium with fusion medium (DMEM GlutaMax, 2% HS, and 1% Penicillin/Streptomycin). To avoid disturbing the formation of the myotubes during differentiation, the medium was replaced only once on the second day of differentiation (with fusion medium supplemented with 3.7 mg/mL Glucose). The cells were allowed to fuse for 5 days and the myotubes were treated as previously described [18] with purified agrins (2 nM) or indicated volume of conditioned media or WCE. The AChRs were labeled with Alexa Fluor 488 conjugated α-bungarotoxin and observed with a X20 or a X40 objective lens under an Olympus IX70 inverted microscope (Olympus Europa, Hamburg) linked to a CDD camera (Princeton Cool SNAP Fx, Trenton, NJ). The number of clusters was evaluated in randomly selected fields with an ImageJ software.

### hiPSC cell culture and differentiation

hiPSC lines from patient 1 and from healthy patients were generated from peripheral blood mononuclear cells (PBMCs), respectively, in the iPSC core facility of Nantes University and in Phenocell®. The PBMCs of Patient 1 were obtained from the DNA and Cell Banks of Généthon and used to derive the 5851-1 hiPSC clone. PBMCs were reprogrammed by Sendai viruses expressing Oct4, Sox2, Klf4, and c-Myc (CytoTune™-IPS 2.0 Sendai Reprogramming kit, Life Technologies and [30]). Briefly, the 5851–1 iPSC clone was picked and expanded on mouse embryonic fibroblasts (MEFs) feeder cells in KSR-FGF2 medium (DMEM/F12 supplemented with 0.1% *β*-mercaptoethanol, 20% knockout serum replacement, 10 ng/mL basic fibroblast growth factor, 2 mmol/L l-glutamine, and 1% NEAA). Until P10, colonies were mechanically passaged with a needle. At P10, iPSC clones were adapted to feeder-free culture conditions: stem cell-qualified Matrigel-coated plates (0.1 mg/mL; BD Bioscience) with IPs Brew XF medium (StemMACS™, Miltenyi Biotec). Feeder-free iPSCs were passaged using the Passaging Solution XF (StemMACS™, Miltenyi Biotec).

As control, three distinct hiPSC lines were used: two hiPSC lines derived from healthy females were used as previously described [29] and one human embryonic stem cell line (SA01) obtained from Cellartis. Experimental protocols were approved by the French Minister of Health (2019-A02599-48). Human embryonic stem cell (hESC) line was used following the recommendation of the French Law of Bioethics and declared at the French Agency of Biomedicine (Number SASB1020178S). Control hiPSC lines were thawed and manually expanded over five supplementary passages. For manual passaging, StemPro EZPassage tool (Thermo Fisher Scientific) was used. The automated cell culture system CompacT SelecT (Sartorius, Gottingen, Germany) was then used to generate a working cell bank using 0.25 mM EDTA (Thermo Fisher Scientific) in PBS without calcium or magnesium for cell passaging. Finally, cells were dispensed into cryovials using the automated system Fill-It (Sartorius) and frozen using CryoMed Controlled-Rate Freezer (Thermo Fisher Scientific). Quality controls (mycoplasma detection, pluripotency marker expression, and genomic integrity) were performed before and after amplification.

Human iPSC-derived MNs were generated as previously described by Maury et al., 2015 [29]. Human iPSCs were dissociated with Accutase (Thermo Fisher Scientific) and resuspended in medium-containing DMEM-F12 Glutamax/Neurobasal (1:1 ratio; Gibco), N2 supplement/B27 without vitamin A supplement (1:2 ratio; Gibco), β-mercaptoethanol (0.1%; Gibco), Penicillin/Streptomycin (0.1%; Gibco), supplemented with small molecules including ascorbic acid (0.5 µM; Sigma-Aldrich), SB431542 (20 µM; TOCRIS-BioTechne, Minneapolis, MN, USA), LDN193189 (0.2 µM; Miltenyi Biotec), CHIR99021 (3 µM; Miltenyi Biotec), and Y-27632 (10 µM; STEMCELLS Technologies, Vancouver, Canada). Cells were seeded in suspension into T25 flask (Dutscher, Bernolsheim, France) to form embryoid bodies (EBs). During the entire culture process, small molecules were added at different time points including retinoic acid (0.1 µM RA; Sigma-Aldrich), Smoothened Agonist (0.5 µM SAG; STEMCELLS Technologies), Brain-Derived Neurotrophic Factor (10 ng/mL BDNF; PreproTech, Rocky Hill, NJ, USA), and *γ*-secretase inhibitor (10 µM DAPT; STEMCELLS Technologies). Then, EBs were dissociated at DIV 10 (days in vitro) and hMNs progenitors were generated. Cells were finally dispensed into cryovials and freezed using CryoMed Controlled-Rate Freezer (Thermo Fisher Scientific).

The co-cultures between hiPSC-derived motoneurons and human skeletal muscle cells were performed as previously described [9]. Briefly, human primary myoblasts (CHQ), from quadriceps muscle biopsy of a 5-day-old infant, were obtained from the MyoBank, Institute of Myology (Paris, France) [11] and were seeded onto 96-well plate and incubated over 24 h at 30,000 cell/cm^2^. The myogenic induction medium was replaced with co-culture medium which was a mix between 1:3 of myogenic differentiation medium and 2:3 of hMNs growth medium. The myogenic differentiation medium was composed of DMEM high glucose GlutaMAX (Gibco), gentamicin (1%; Gibco), and insulin (10 µg/mL; Gibco). The hMNs growth medium was composed of a basal medium which is a mix between DMEM-F12 Glutamax/Neurobasal (1:2 ratio; Gibco), N2 supplement/B27 no vitamin A supplement (1:2 ratio; Gibco), *β*-mercaptoethanol (0.1%; Gibco), and Penicillin/Streptomycin (0.1%; Gibco), supplemented with small molecules including ascorbic acid (0.5 µM; Sigma-Aldrich), retinoic acid (0.1 µM; Sigma-Aldrich), Smoothened Agonist (0.5 µM SAG; STEMCELLS Technologies), Brain-Derived Neurotrophic Factor (10 ng/mL BDNF; PreproTech), Glial-Derived Neurotrophic Factor (10 ng/mL GDNF; PreproTech), and γ-secretase inhibitor (10 nM DAPT; STEMCELLS Technologies). Y-27632 (10 µM; STEMCELLS Technologies) was used only for the thawing. The hMNs’ progenitors generated in 10 days following the previous protocol were seeded at a concentration of 60,000 cells/cm^2^ and plated directly over the myoblasts and incubated at 37 °C with 5% CO_2_ for up to 4 days to mature and differentiate.

### In vitro immunofluorescence

Primary MNs or cell line cultures were fixed in 4% PFA for 15 min at room temperature, washed 3 times in PBS, and blocked and permeabilized in PBS with 0.1% Triton X-100 and 3% BSA. The primary antibody was applied overnight at 4 °C diluted in blocking buffer, washed 3 times in PBS with 0.1% Triton X-100, the secondary antibody for 2 h at room temperature, with DAPI, and eventually WGA, then washed four times in PBS with 0.1% Triton X-100, and mounted in Vectashield® (H-1000, Vector Laboratories) or FluoroMount (#00-4958-02; Thermo Fisher Scientific). Fluorescence was observed under either an EVOS M5000 imaging system (Thermo Fisher Scientific), Zeiss AxioImager Z1 standard microscope, a Leica SP5 confocal spectral microscope, or Zeiss LSM800 confocal microscope. To quantify subcellular co-localization, at least 15 images per condition were taken randomly at 63 × objective using a confocal microscope setup on an equivalent scan thickness by adapting the pinhole between channel wavelengths. Acquistion were repeated at least three times from independent experiments. Images were analyzed using Metamorph (Molecular devices) software using scripts edited as follow. Multi-TIFF images were split in two channels such as agrin versus Golgi apparatus marker for example. A threshold was defined manually in the Metamorph script for each channel to select the staining area, then the area or the intensity value overlapping the two channels was measured using the builtin function “Measure Colocalization” of Metamorph. Data from the three experiments were pooled and analyzed using the Prism software. To quantify apoptotic SHEP cells, we acquired at objective × 10 (EVOS microscope), at least 20 images per condition, in duplicated wells, for each of three independent experiments. Cell counting were automated using the MetaMorph application “Multi Wavelength Cell Scoring” which allows determining the number of cells in the images by the DAPI channel, the eGFP-positive cells in the green channel, and among them the activated Caspase-3-positive cells in the red channel. To do so, we determined the following cell staining parameters for each channel: staining approximate minimum width, maximum width, intensity above local background, and minimum stained area. Collected mean data for each image were pooled by well in excel software. Then, statistics and plots were done in Prism software.

hiPSC-derived MNs were fixed with 4% PFA for 15 min and further permeabilized and blocked with Triton X-100 (0.1%; Sigma-Aldrich) and BSA (2%; Gibco) in PBS for 30 min. Primary antibodies were then added and incubated at 4 °C overnight in PBS/BSA/Triton solution. Appropriate Alexa Fluor 488/594/647-conjugated secondary antibodies (1:1000; Invitrogen) were used with 4′,6-diamidino-2-phenylindole (DAPI) nuclear counterstain (1:1000; Invitrogen) for 2 h at room temperature. Staining was visualized and imaged acquired using ImageXpress micro imaging system (Molecular Devices^®^) and was analyzed using MetaXpress software. AChR clusters’ morphometric analyses were performed using Fiji Software and using an exclusion threshold of 5 µm^2^.

### Antibodies

The rabbit polyclonal anti-human agrin (1:5000) was kindly provided by Stephan Kröger (Planegg-Martinsried, Germany) and used at 1:5000 for immunofluorescence (IF) and at 1:10,000 for western blotting (WB) [10, 14]. And the following commercial antibodies were used in this study: mouse monoclonal anti-NF-M (#2H3, DSHB); mouse anti-KDEL (clone 10c3; NBP1-97469; Novus Biologicals; for IF 1:400); rabbit polyclonal anti-GM130/GOLGA2 (#NBP2-53420; Novus Biologicals; for IF 1:400); rabbit polyclonal anti-cleaved Caspase-3 (#9661; Cell Signaling Technology; for IF 1:400); rabbit polyclonal anti-GRP78/BIP (#11587–1-AP; Proteintech; for WB 1:3000); mouse monoclonal anti-Tuj1 (# 801213; BioLegend; for IF 1:1000); goat polyclonal anti-Islet1 (#GT15051; Neuromics, for IF 1:200); mouse monoclonal anti-Islet1/2 (#39,4D5; Developmental Studies Hybridoma Bank, DSHB; for IF 1:50); mouse monoclonal anti-heavy chain of myosin II, MF20 (# 14-6503-82; ThermoFisher Scientific; for IF 1:500); the clusters of AchRs were labeled with Alexa Fluor 488/555-conjugated α-bungarotoxin (#B13422 or #B3545; Thermo Fisher Scientific; for IF 1:1000) or with mouse anti-acetylcholine receptor nicotinic alpha 1 subunit in hiPSC (#mAb35, DHSB); horseradish peroxidase-coupled secondary antibodies, sheep anti-mouse (#NA931V; for WB 1:1000), and sheep anti-rabbit (#NA934V; for WB 1:1000) antibodies were from GE Healthcare. For fluorophore-coupled secondary antibodies, donkey anti-rabbit TRITC (#711-025-152; Jackson Immuno Research; for IF 1:1000) or goat anti-guinea pig TexasRed (#106-076-003; Jackson Immuno Research; for IF 1:1000) or donkey anti-mouse Alexa Fluor 488/555/647 (# A-21206, # A3279 and # A-31573 respectively; ThermoFisher Scientific; for IF 1:1000) were used; 4′,6-diamidino-2-phenylindole (DAPI) was used for nuclear counterstain (Invitrogen; 1:1000). Finally, to quantify agrin proteins on a ChemiDoc imager (Bio-Rad), fluorescent secondary antibodies anti-rabbit (#12005870 and #12005870; StarBright, Bio-Rad; for WB 1:5000) were used.

### RT-PCR and RT-qPCR

Total RNAs were extracted with RNeasay Plus Mini kit (#74134; Qiagen) according to the manufacturer’s recommandations. RNAs were reverse transcribed with random hexamers primers by RevertAid™ Reverse Transcriptase (#EP0441; Fermentas Life Science). RT-qPCR was performed using SYBR Green Mastermix (Qiagen) in the CFX-connect system (Bio-Rad). Relative expression levels were normalized to GAPDH, HPRT, and TBP house-keeping genes expression using the ΔΔ*C*_*t*_method. And to measure the transcript levels of sXBP1 (spliced XBP1), an indicator of endoplasmic reticulum (ER) stress, RT-PCR band intensities were analyzed by densitometry, and the levels of sXBP1 and uXBP1 (unspliced XBP1) relative to total XBP1 (uXBP1 + sXBP1) were calculated as described in Bouchecareilh et al. [4] and in Yoon et al., [48]. PCR products were resolved by a 5% agarose gel electrophoresis (# 10975–035; UltraPure™ Agarose-1 000; Invitrogen) and visualized using ethidium bromide and UV transillumination.

Sets of gene-specific primers used were as follows in Table 1.

**Table 1:** Oligonucleotide primers for PCR analysis

Gene	Primer sequence	
	Forward primer (5′–3′)	Reverse primer (5′–3′)
GAPDH	GAAGGTGAAGGTCGGAGTC	GAAGATGGTGATGGGATTC
HPRT	GACACTGGCAAAACAATGCA	GGTCCTTTTCACCAGCAAGCT
TBP	CACGAACCACGGCACTGATT	TTTCTTGCTGCCAGTCTGGAC
u/sXBP1	GGTCTGCTGAGTCCGCAGCA	AAGGGAGGCTGGTAAGGAAC
uXBP1	CAGACTACGTGCACCTCTGC	CTGGGTCCAAGTTGTCCAGAAT
sXBP1	GCTGAGTCCGCAGCAGGT	CTGGGTCCAAGTTGTCCAGAAT
tXBP1	TGAAAAACAGAGTAGCAGCTCAGA	CCCAAGCGCTGTCTTAACTC
GRP94	GGGTGTGGTGGACTCAGATG	ACGTGTTCGATTCGAGTGGA
GRP78	CACTCCTGAAGGGGAACGTC	TTTCAACCACCTTGAACGGC
CHOP	AGGCACTGAGCGTATCATGT	CTTGAACACTCTCTCCTCAGGT
GADD34	GCCCAGAAACCCCTACTCAT	CCCAGACAGCCAGGAAATG
EDEM1	ATTTCGAAGCATGTGGGAGC	GCTCATTTCGGGAAGCTTCT

### Statistical analysis

Each experiment was repeated at least three times independently. Data were analyzed with Excel (Microsoft) or Prism (GraphPad). First, data distribution was analyzed by D'Agostino and Pearson omnibus normality test for Gaussian distribution and Bartlett's test for equal variances. Then, data from two conditions following Gaussian distribution or equal variance were analyzed by Student’s *t* test. Data from several conditions each showing normality and equal variance were compared with one-way ANOVA test followed by Dunnett's multiple comparison test. Otherwise, data that do not show a Gaussian distribution or an equal variance were analyzed with non-parametric Kruskal–Wallis test followed by Dunn's multiple comparison test. Finally, a two-way ANOVA was used to estimate the difference in the mean of data according to two categorical variables.

## Results

### Clinical features of 3 patients from 2 families and electrophysiological and morphological evidence for the diagnosis of CMS

#### Family 1 (Patient 1)

Family 1 is a non-consanguineous French family from which both parents and the brother of the female patient are unaffected.

**Fig. 1:**
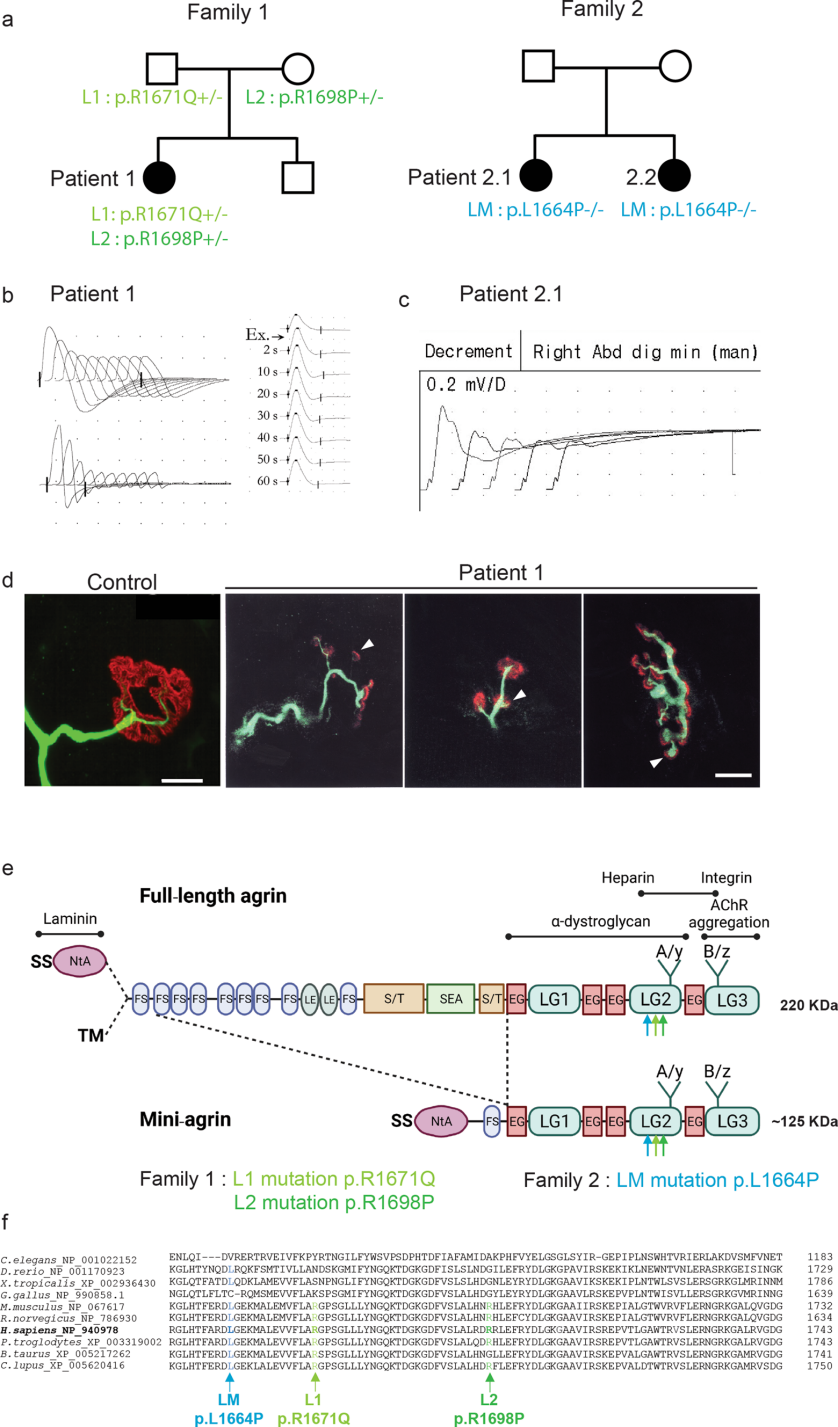
Mutations in *AGRN* Gene Causing CMS. **a** Family 1 and 2 pedigrees. **b**, **c** EMG reveals obvious decremental response of the compound muscle-fiber action potential (CMAPs) in Patient 1 and Patient 2.1. **d** Whole-mount preparations of muscle biopsy stained with α-bungarotoxin in red and stained for axon terminals with neurofilament in green. In control, the axonal branch classically ends as a fork and innervates a well-defined synaptic structure. In Patient 1, the neurofilament staining showed frequent presynaptic sprouting (arrowhead). Scale 10 µm. **e** Domain organization of agrin and recombinant mini-agrin used in this study and position of the L1, L2 and LM mutations. Agrin is a mosaic protein composed of the following domains: SS, signal peptide; NtA, N-terminal laminin-binding domain, red; TM, trans-membrane domain; FS, follistatin-like, blue; LE, laminin EGF-like, orange; S/T, serine/threonine-rich region, yellow; SEA, Sea urchin sperm protein, Enterokinase and Agrin motif, purple; EG, EGF-like, red; LG, laminin-globular-like, green; LG2 domain interacts with α-Dystroglycan and integrins; Sites of alternative splicing: SS-NtA or TM at the 5’end, A/y in the LG2 domain, B/z in the LG3 domain. **f** Multiple alignments of amino acid sequences in the agrin LG2 domains (residues 1949–1743 from H. sapiens_NP_940978) show the positions of L1, L2 and LM mutations

The index Patient 1 (Fig. 1a) was born without family medical history and has reported having been unable to run since early childhood and having had ocular pursuit and pupillary reactivity problems. At the age of 6 years, weakness, fatigability, hypotonia, and scoliosis were noted. At the age of 14 years, clinical evaluation and electromyography were suggestive of spinal muscular atrophy (SMA). At the age of 18 years, proximal and distal muscular weakness was reported in all 4 limbs, but no ocular nor bulbar deficiency. At the age of 22 years, a biopsy was performed in the deltoid muscle. Histopathologic analysis revealed high heterogeneity in muscle-fiber size, with small angular fibers some of them showing grouping, and some with central nuclei. Trichrome staining revealed no overload or interstitial lesion. Atrophic fibers were preferentially type I. Glycogen and lipids were normally distributed (neither representative images nor the biopsy tissue are yet available). Altogether, the histopathologic analysis showed limited abnormalities in favor of a denervation–reinnervation process compatible with SMA. At the age of 30 years, proximal muscle atrophy in arms and legs became obvious and the patient developed respiratory muscle dysfunction, requiring nighttime respiratory support. At the age of 39 years, the patient required permanent ventilator assistance and tube feeding. At this time, a new repetitive nerve stimulation (RNS) test was performed and showed an obvious decremental response of the compound muscle-fiber action potential (CMAPs) at the 4 limbs (Fig. 1b). Surprisingly, clear and reproducible post-exercise facilitation was also observed, indicating presynaptic involvement such as in the Lambert–Eaton myasthenic syndrome. Tests to detect serum anti-AChR antibodies were negative, and the patient responded favorably, though incompletely, to anticholinesterase medications and to 3,4-diaminopyridine. The muscle biopsy showed abnormal morphology of neuromuscular junction (NMJ) with frequent presynaptic sprouting (Fig. 1d).

#### Family 2

Family 2 is a Polish family with two affected sibling patients (Patient 2.1 and Patient 2.2) and where the parents are unaffected. The two sisters share a similar severe phenotype suggesting an SMA-like type 0 and were the product of complicated pregnancies and birth, as well as having abnormal motor milestones.

Patient 2.1 was (Fig. 1a) born by C-section in the 35th week of gestation after the pregnancy was complicated with polyhydramnios, practically no fetal movements and opaque amniotic fluid. Her Apgar scores were 1-3-5 points, there was a lack of sucking reflexes, and she failed to initiate effective respiratory action. Her birth weight was 2750 g. She spent the first 6 weeks of her life in the hospital: she was floppy, had little spontaneous activity, was tube-fed, and required full-time ventilation. When she was 4 months old, she required percutaneous endoscopic gastrostomy (PEG). When she was approximately 12 months old, she had some spontaneous movements in the upper extremities distally and elbow flexion. She subsequently lost this ability. During infancy, multiple diagnostic tests were performed: SMA and Prader–Willi syndrome were excluded. Karyotype and array comparative genomic hybridization (aCGH) were normal. Treatment with 3,4-DAP, salbutamol, and ephedrine was attempted, but did not provide benefit. At the last examination at the age of 6 years, she is treated with Mestinon with slight improvement—according to the parents, she has better eyelid mobility but remains completely paralyzed. The girl communicates with single words and with the movements of her eyelids. Her neurological exam showed: PEG, respirator (tracheostomy), bilateral ptosis *R* > *L*, limitations of eye movements, and high-arched palate. Severe global muscle atrophy was observed. Absent deep tendon reflexes (DTR), minimal range of voluntary movements distally in upper and lower extremities, and only trace of muscle contraction in the muscles of her arms were noted. Like Patient 1, RNS investigation on Patient 2.1 showed obvious decremental response of the CMAPs at the four limbs (Fig. 1c).

Patient 2.2 shares similar clinical features with her sister Patient 2.1. She is a 3-year-old girl, born by C-section after 37 weeks’ gestation, and the pregnancy was complicated with polyhydramnios and poor fetal movements. Her Apgar scores were 1-3-3-4, there was no sucking reflex, and she was unable to initiate effective breathing action on her own. Her body weight was 2520 g. Similar to her sister, she was tube-fed from birth and started using a PEG at 2 months of life and has been ventilated full time from birth. She has preserved a small range of movements in hands and arms up to the elbow level. Treatment was attempted with Firdapse, salbutamol, and ephedrine without major benefit. She tolerated Mestinon poorly in a dose of 2 × 24 mg (diarrhea). On examination at age 3 years: PEG, respirator (tracheostomy), eyes almost closed, restriction of eye movements, and high-arched palate were observed. She had global muscle atrophy, absent DTR, minimal range of motion distally in upper and lower extremities, and traces of movement in the elbow and knee joints. She has flexion contractures of the third fingers of both hands.

### Molecular finding in *AGRN* gene

At first, clinical presentation of the 3 patients were suggesting an SMA-like disease, and *SMN1* gene mutation was systematically excluded. The appearance of a decremental response at EMG in patient 1 at the age of 30 prompted us to investigate mutations in CMS causing genes. Based on the pattern of inheritance, candidate genes with homozygous or two heterozygous variations were searched in AChR subunits genes, *MUSK, RAPSN* (RAPSYN), *LRP4, DOK7, SCN4A, LAMB2* (Lamin β2), *COLQ*, and *AGRN* (Agrin). Two rare compound heterozygous variants in *AGRN* exon 29 were identified (reference sequence NM_198576.3): c.5012G > A and c.5093G > C, that lead to non-synonymous substitutions, p.R1671Q and p.R1698P, relatively to Uniprot O00468-1 agrin protein sequence. Both mutations are located in the second Laminin-Globular domain LG2 (interpro IPR001791) of agrin (Fig. 1e, f). Thereafter, these mutations will, respectively, be referred to as L1 and L2. The L1 c.5012G > A allele is present in the gnomAD database of control individuals (ID:rs769667244), at a low frequency (2.906E−5), but never in homozygous state. The L2 c.5093G > C allele is not found in gnomAD, although four heterozygous variants exist that change the 1698 arginine residue to amino acids other than proline at a low frequency (Gly at 8.07E−6, Ser at 8.07E−6, Cys at 1.21E−5, and His at 3.23E−5) and never in homozygous state. Analysis of the parent’s DNA confirmed autosomal recessive inheritance (Fig. 1a): each parent carries one of the mutations and both are asymptomatic. Both mutations, therefore, probably cause a loss of function, which is compensated in the presence of a normal allele.

Whole Exom Sequencing and Sanger sequencing performed on Patient 2.1 at age 4 years and her younger sister Patient 2.2 at 1 year of age, confirmed CMS with a homozygous mutation c.4991 T > C in the *AGRN* gene. This mutation, which is absent from gnomAD, leads to a non-synonymous substitution p.L1664P referred to in the text as mutation LM (Fig. 1a). To summarize, the 3 missense mutations L1, L2 and LM are located in the same LG2 domain of agrin and affect an amino acid well conserved during evolution (Fig. 1f).

### L1, L2 and LM mutations interfere with agrin production by motoneurons

**Fig. 2:**
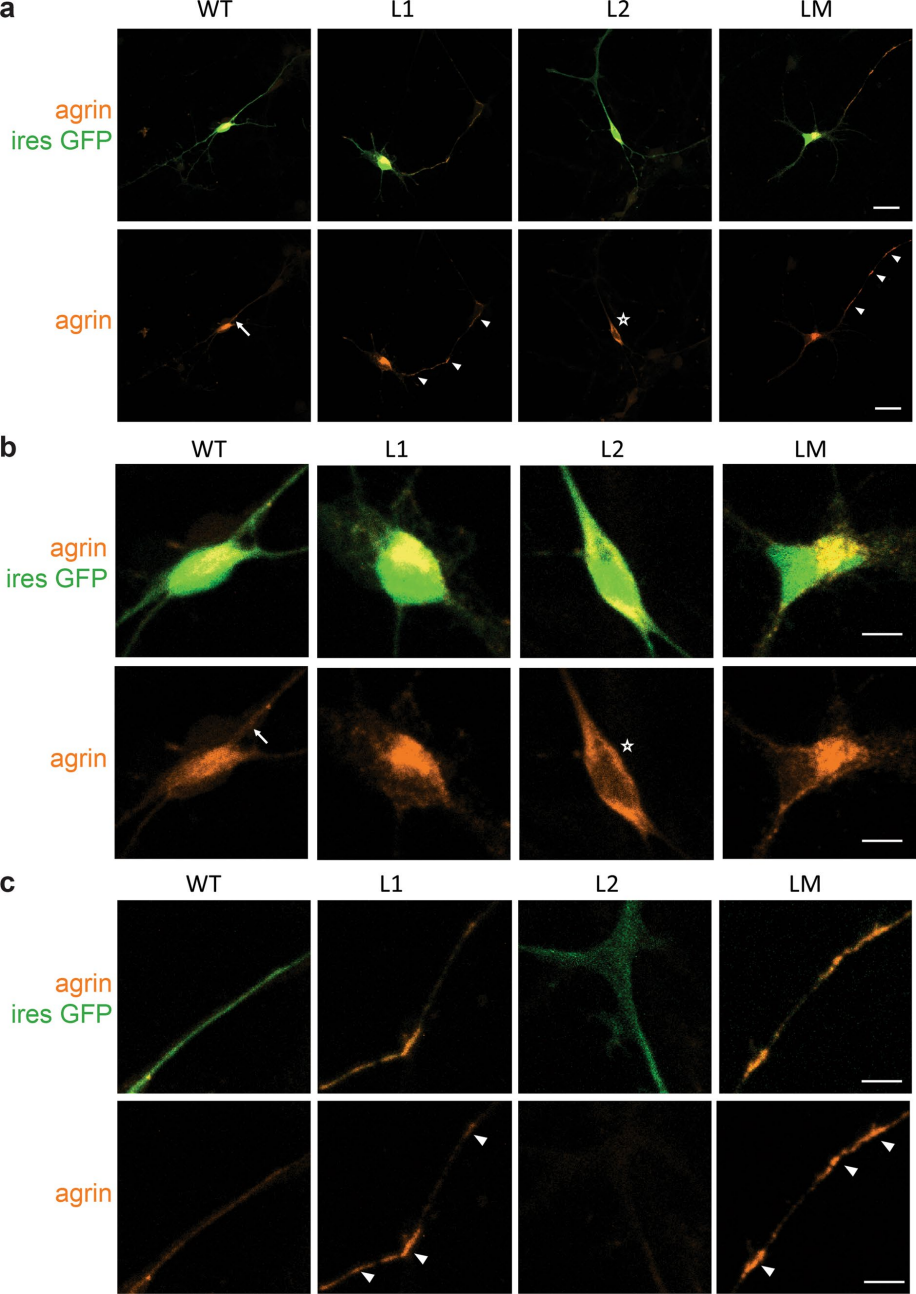
Abnormal subcellular localizations of mutant mini-agrin in primary motoneurons. **a** Representative confocal images of MNs 2 days after transfection with WT, L1, L2 or LM mini-agrin IRES eGFP (green) constructs. Overexpressed human agrins were revealed with an anti-human-agrin antibody (red). **b** Magnification of the cell bodies from images in **a**. WT agrin showed a diffuse distribution often with stronger labeling at the soma/neurite boundary characteristic of secreted proteins (arrow). L2 agrin accumulates exclusively in the soma (star). **c** Magnification of the axonal projections from images in **a**. L1 and LM agrin accumulate along the axo-dendritic compartment (white arrowhead). Scale bars: 20 μm

To evaluate the effect of mutations in the *AGRN* gene, mini-agrins that recapitulate agrin functions both in vivo and in vitro are classically used. Human WT and mutant *AGRN* constructs were transfected in primary MNs purified from mouse spinal cords. To visualize transfected MNs, *AGRN* cDNAs were inserted in a bicistronic vector containing an internal ribosomal entry site that allowed the simultaneous expression of human mini-agrin and eGFP (Fig. 2). Transfected MNs expressing either WT, L1, L2 or LM agrins displayed similar morphologies, typical of MNs with soma, axon and dendrites labeled with SMI32 antibody (data not shown).

Staining with an anti-human agrin antibody that does not recognize mouse agrin (Suppl. Figure 1) showed a diffuse distribution of WT agrin, often with stronger labeling at the soma/neurite boundary, characteristic of secreted proteins (Fig. 2b) [12]. In contrast, L1 and LM agrins accumulated in dots along the axo-somatic and dendritic compartments, and L2 agrin exclusively accumulated in the soma and was excluded from axon and dendrites (Fig. 2c).

To determine whether axonal accumulations of agrin were inside or outside axons, agrin immunostaining was performed on non-permeabilized MNs. The SMI32 antibody that recognizes NEFH protein was used as a control of non-permeability. As expected, no SMI32 staining was observed (data not shown) whereas staining was detected with WT or L1 agrins (Suppl. Figure 2a). WT agrin linearly diffused on the plate coating around the axon, whereas L1 agrin concentration exponentially decreased with the distance to the axon (Suppl. Figure 2b), indicating that the diffusibility of secreted L1 agrin is very low.

### L1, L2 and LM mutations induce trafficking defects in vivo

**Fig. 3:**
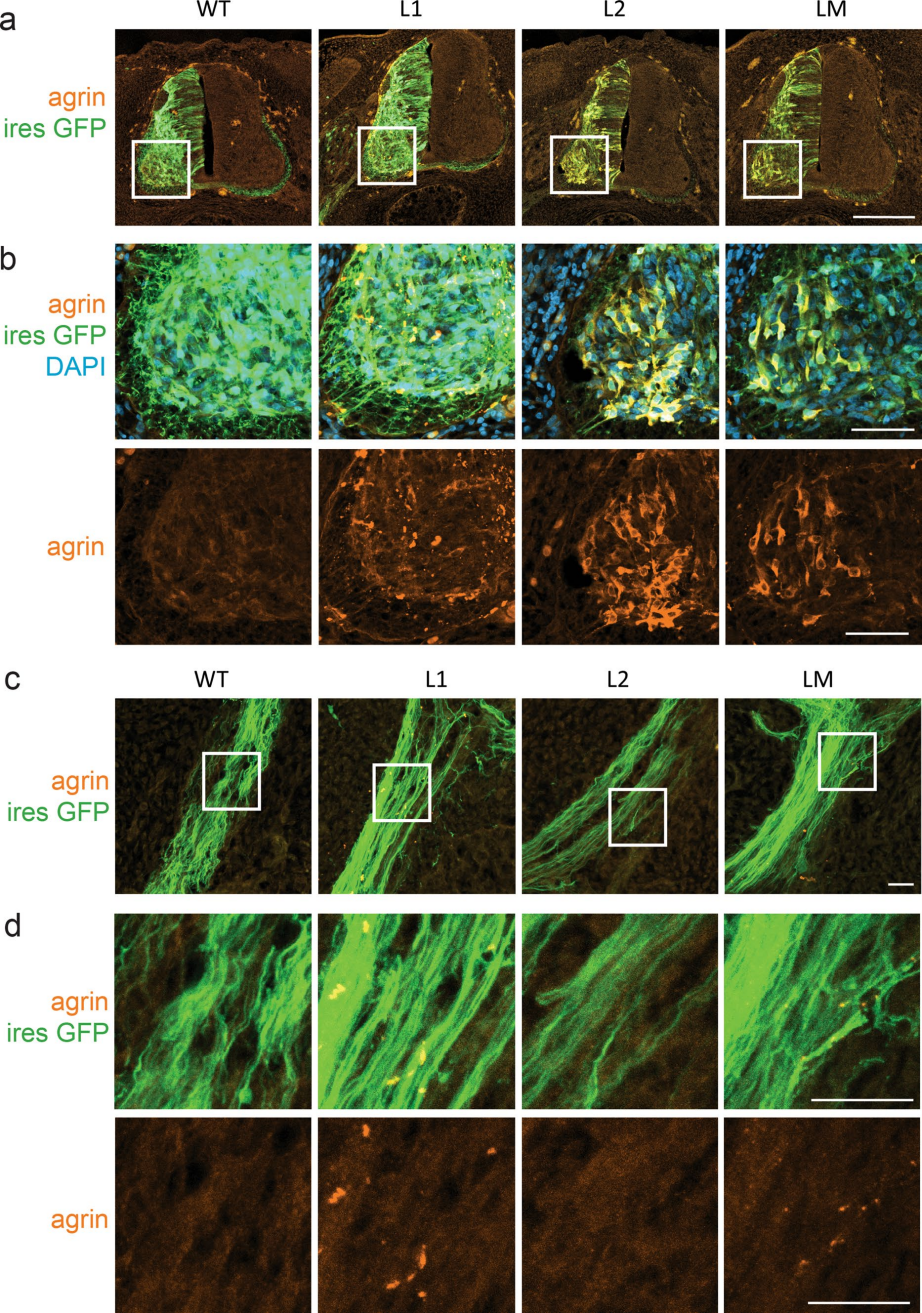
Mutant mini-agrin are mislocalized in motoneurons in vivo. **a** Confocal images of spinal cord cryosection from chick neural tube electroporated with WT, L1, L2 or LM mini-agrin IRES eGFP constructs. Electroporated neurons are identified by eGFP (green), and human agrin immunostaining (red). Nuclei are stained with DAPI (blue). Scale bar: 200 µm. White boxes show the enlarged region. **b** Higher magnification on the anterior horn shows the accumulation of the three mutant agrins in the soma of MNs. Scale bar: 50 µm. **c**, **d** Confocal images of axonal projections from electroporated motoneurons in the upper limb revealed by eGFP show focal accumulations of L1 et LM but not L2 mutants along the axon projections. Scale bar: 20 µm. White boxes show regions enlarged in **d**

To investigate agrin distribution in vivo, bicistronic expression vectors co-expressing agrin and EGFP were electroporated in the neural tube of chick embryos at stage HH14-15. Electroporation time was chosen, so that 2 days after electroporation, neuronal progenitors have departed from the ventricular zone of the neural tube, have become postmitotic, and have differentiated into mature MNs growing axons. At this stage (HH24-26), spinal MNs were analyzed by immunofluorescence on embryo cryosections using confocal microscopy (Fig. 3).

Analysis of eGFP distribution indicated that electroporation of the agrin constructs did not alter the neuronal morphology or, more generally, spinal cord and nerve development. Motoneurons were correctly positioned in the anterior horn and axon projections toward limb buds were normal (Fig. 3a), consistent with the observation that none of the mutations altered the morphology of mouse primary MNs (Fig. 2). Proper MN differentiation was confirmed by Islet1/2 staining. In embryos electroporated with mutant agrins, the distribution of Islet1/2-positive nuclei in the ventral horn was similar to that of eGFP-electroporated and non-electroporated embryos (Suppl. Figure 3).

Analysis of human agrin distribution in the anterior horn with an anti-human agrin antibody indicated that WT agrin did not accumulate at specific sites, consistent with its secretion at the axon tip (Fig. 3). In contrast, all three mutant agrins accumulated in the soma of MNs in the anterior horn (Fig. 3b). In addition, L1 and LM mutants generated focal accumulations along the projecting axons (Fig. 3c, d). As observed in cultured MNs, L2 agrin was exclusively detected in the soma of electroporated MNs (Fig. 3b). Consistently, no L2 agrin was observed in the axons (Fig. 3c, d).

Altogether our in vitro and in vivo results suggested that the pathogenic effect of these mutations could reside in the inability of MNs to produce enough mutant agrin at the NMJ.

### Mutant agrins are poorly secreted and are retained in the endoplasmic reticulum

**Fig. 4:**
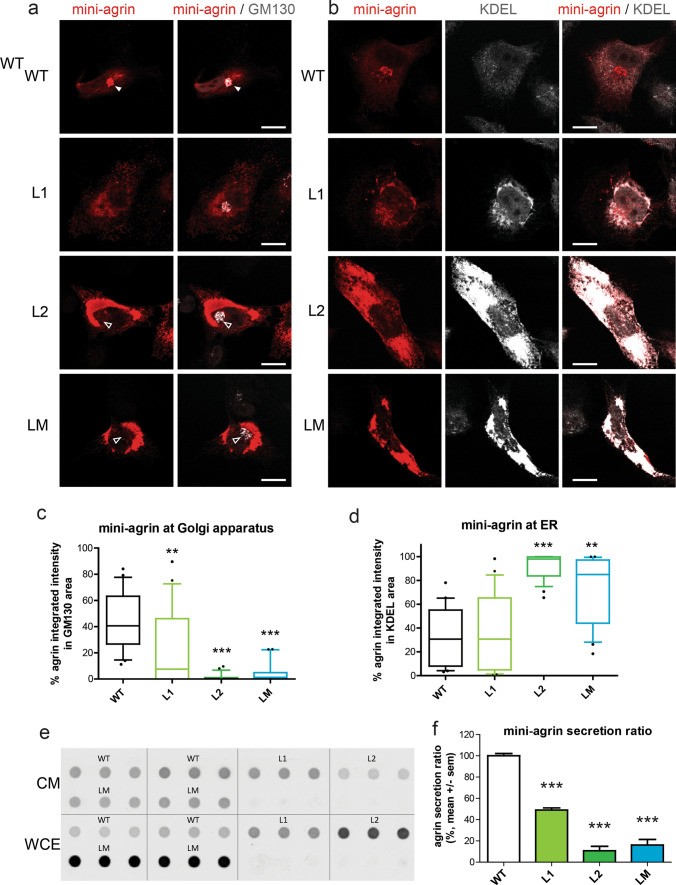
Mutant mini-agrins accumulate in the endoplasmic reticulum and are less secreted. **a** Confocal images of SHEP cells transfected with agrin IRES eGFP (green) counterstained for agrin (red) and Golgi apparatus (GM130, white). Full white arrowheads show agrin enrichment at the Golgi apparatus. Empty white arrowheads indicate the absence of agrin in the Golgi apparatus. Scale bar: 10 μm. **b** Confocal images of SHEP cells transfected with agrin IRES eGFP counterstained for agrin and an endoplasmic reticulum marker KDEL (white). Scale bar: 10 μm. **c**, **d** Quantification of agrin/Golgi co-localization and agrin/endoplasmic reticulum (ER) co-localization (Kruskal–Wallis test followed by Dunn's multiple comparison test; **p < 0.01; ***p < 0.001). **e** Representative dot-blot for agrin quantification in conditioned medium (CM) and whole-cell extract (WCE) from cultures expressing WT, L1, L2 or LM mini-agrins. **f** Graphic representation of the ratio of secreted agrin quantified from the dot-blots and at least three independent experiments (one-way ANOVA followed by Dunnett’s test; ***p < 0.001)

Conventional protein secretion is the trafficking route that secretory proteins undertake when they are transported from the endoplasmic reticulum (ER) to the Golgi apparatus, and subsequently to the plasma membrane via secretory vesicles or secretory granules [44]. Endoplasmic reticulum (ER) and Golgi apparatus were visualized in transfected the SHEP neuroblastoma cell line using the KDEL motif and GM130 labeling, respectively. As expected for a secreted glycosylated protein, WT agrin was mostly visualized in the Golgi apparatus where it undergoes maturation before secretion (Fig. 4a). The accumulation of all mutant agrins in the Golgi apparatus was reduced, and prominent somatic staining evocative of the ER was present (Fig. 4a). Colocalization with KDEL staining consistently revealed an increase in the levels of mutant agrins in the ER compared to WT agrin (Fig. 4b). This was most striking with the L2 mutant that was never present in the Golgi apparatus (Fig. 4c) and induced a very strong increase of the ER volume (Fig. 4d). Altogether, this suggested that mutant agrins did not reach the Golgi apparatus properly after their translation in the ER, and therefore could not be efficiently secreted. To confirm this, the levels of agrin retained in the cells and secreted in the culture medium were evaluated in a dot-blot. As shown in Figs. 4e and 4f, the ratio between secreted and intracellular agrin was strongly diminished for all mutants compared to WT agrin.

**Fig. 5:**
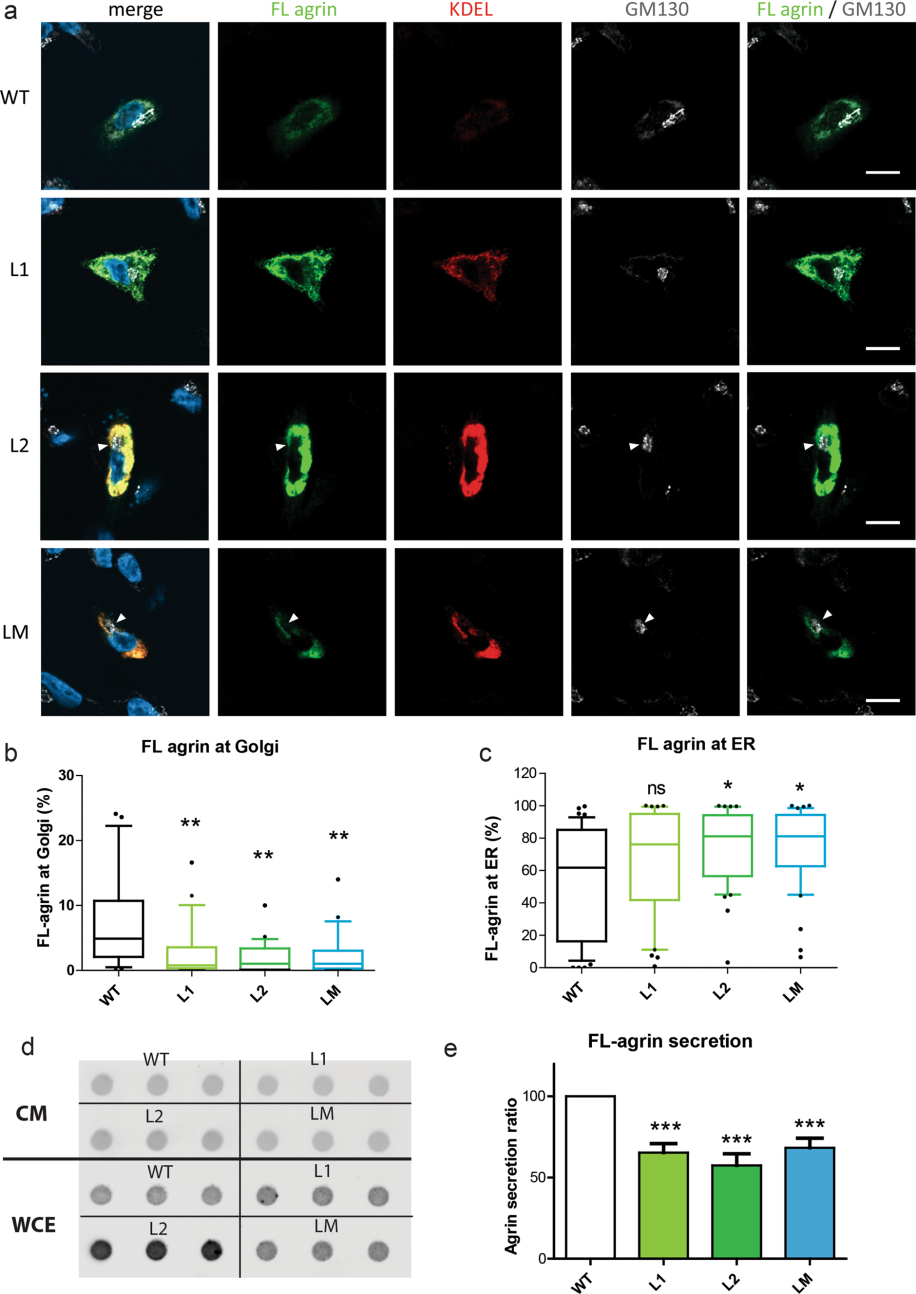
Mutant FL-agrins accumulate in the endoplasmic reticulum and are less secreted. **a.** Confocal images of SHEP transfected cells with FL-agrin fused to eGFP (in green), counterstained with the endoplasmic reticulum marker KDEL (in red) and the Golgi apparatus marker GM130 (in white). White arrow shows agrin exclusion from the Golgi apparatus. Scale bar: 10 μm. **b** Quantification of agrin/Golgi co-localization (Kruskal–Wallis test followed by Dunn's multiple comparison test; **p < 0.01). **c** Quantification of agrin/endoplasmic reticulum (ER) co-localization (Kruskal–Wallis test followed by Dunn's multiple comparison test; *p < 0.05). **d** Representative dot-blot for agrin quantification in conditioned medium (CM) and whole-cell extract (WCE) from cultures expressing WT, L1, L2 or LM FL-agrins. **e** Graphic representation of FL-agrin secreted ratio quantified from the dot-blots and at least three independent experiments (one-way ANOVA followed by Dunnett's multiple comparison test; ***p < 0.001). **c** Quantification of the percentage of FL agrin that colocalized with the Golgi apparatus marker GM130 (Kruskal–Wallis test followed by Dunn's multiple comparison test; **p < 0.01)

To eliminate the possibility that the mutations would only affect mini-agrin secretion, L1, L2 and LM mutations were introduced in a full-length agrin construct fused with GFP at its N-terminal end (FL-agrin). FL-agrin intracellular distribution and secretion were evaluated by immunofluorescence (Fig. 5a). As for mini-agrins, mutant FL-agrins did not reach the Golgi apparatus efficiently (Fig. 5b) and accumulated at the ER (Fig. 5c). Consistently, the ratio between secreted and intracellular FL agrin was diminished for all mutants compared to WT agrin (Fig. 5d-e).

### Mutant agrins show reduced solubility and trigger unfolded protein response

**Fig. 6:**
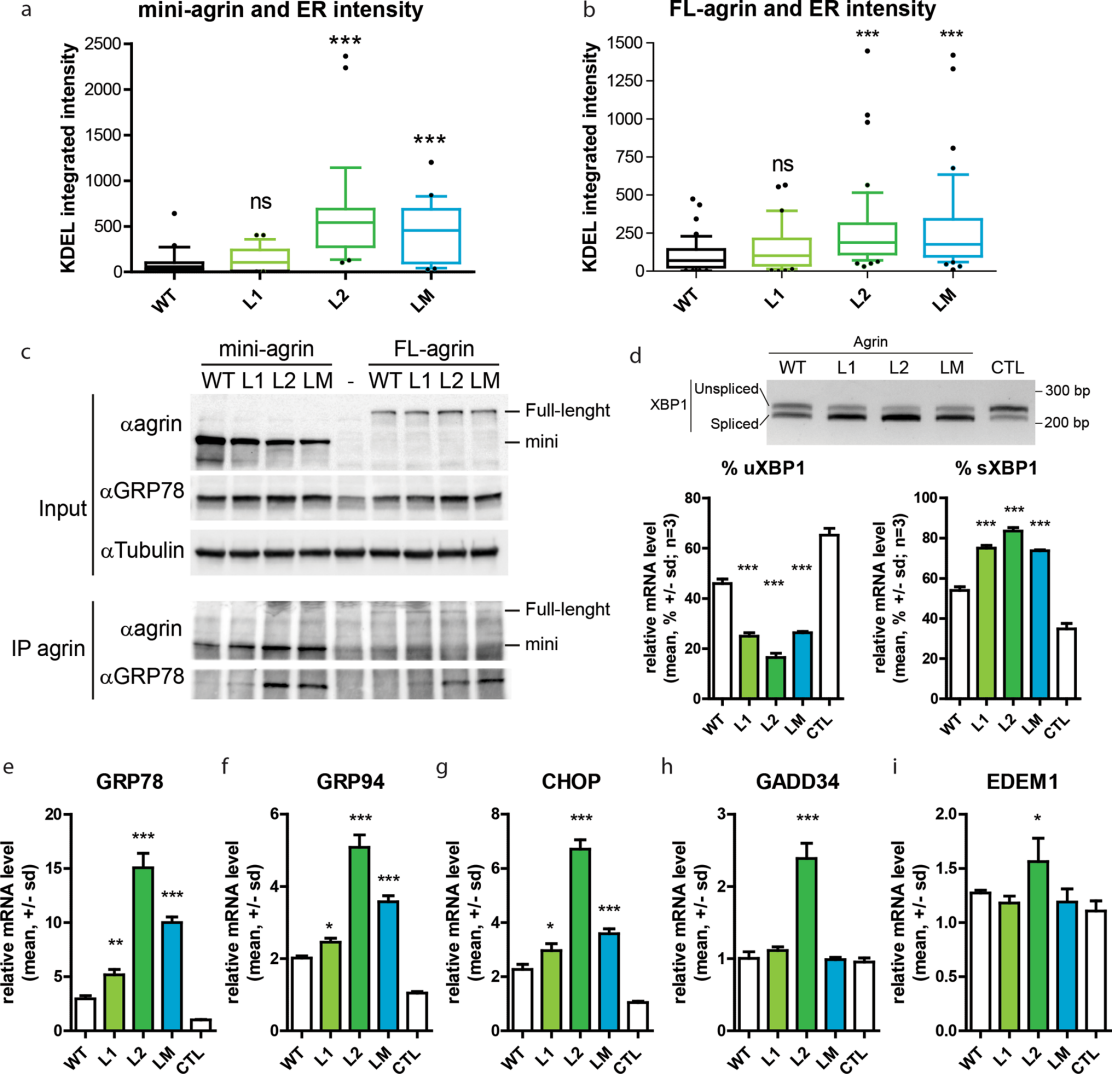
Mutant agrins trigger unfolded protein response. **a**, **b** Quantification of the size and intensity of KDEL immunostaining on SHEP cell confocal images after expression of mini or FL-agrins (*n* = 3; Kruskal–Wallis test followed by Dunn's multiple comparison test). **c** Co-immunoprecipitation of mini and FL-agrins with GRP78/BIP. IP agrin: agrin immunoprecipitation. α indicates the antibody used for the western blot.). **d** Gel electrophoresis resolving the unspliced (uXBP1) and spliced forms (sXBP1) of XBP1 and densitometric analysis of u/sXBP1 band intensities relative to the total (uXBP1 + sXBP1) XBP1, (*n* = 3; one-way ANOVA followed by Dunnett's Multiple Comparison Test). **e**–**i** GRP78, GRP94, CHOP, GADD34, and EDEM1 mRNA quantification by RT-qPCR on HEK293T transfected with WT or mutant mini-agrin expression vectors or empty vector in control (*n* = 3; one-way ANOVA followed by Dunnett's multiple comparison test to compare all mutant agrins to WT agrin; *p < 0.05; **p < 0.01; ***p < 0.001)

Accumulation of unfolded proteins in the ER activates a stress response pathway known as the Unfolded Protein Response (UPR) which is initiated by the binding of the ER chaperone GRP78/BIP to the exposed hydrophobic regions of misfolded proteins. Subsequent steps of the UPR involve ER membrane expansion and accumulation of various ER chaperones containing the ER-localisation motif KDEL. To determine the impact of the mutant agrins on ER volume and enrichment in chaperones, the KDEL motif was immunostained on SHEP cells expressing mini and FL-agrins (Figs. 4b, 5a and 6a,b). In both cases, L2 and LM agrins’ expressions were associated with an increase of KDEL staining consistent with the retention of unfolded proteins. L1 images also showed increased accumulation and co-localization with KDEL (Fig. 4b), but the quantification results were not significant (Fig. 4d). Consistently, GM130 immunofluorescence to visualize the Golgi apparatus showed that L2 and LM mutants did almost not reach the Golgi at all, and that the amount of L1 agrin present in the Golgi was significantly reduced compared to WT agrin (Fig. 4a and Fig. 4c). Similar experiments were performed with FL-agrins and comparable results were obtained (Fig. 5).

To further confirm the induction of ER stress by mutant agrins, binding to the GRP78/BIP chaperone was evaluated by co-immunoprecipitation on HEK293 cells 48 h after transfection with either mini or FL-agrin expression vectors. As expected, L2 and LM agrins strongly co-precipitated with GRP78 chaperone (Fig. 6c). The L1 mutant coprecipiated much less efficiently with GRP78, consistently with the fact that it is less retained in the ER and better secreted that L2 and LM mutants.

To better characterize the UPR branches activated by mutant agrins, activation of target genes of the three arms of the UPR was investigated. Namely, GRP78 and GRP94 as ATF6a target, GADD34 and CHOP as PERK targets and XBP1 and EDEM1 as IRE1a target. At first, XBP1 splicing, an indicator of endoplasmic reticulum (ER) stress, was analyzed. The levels of spliced XBP1 (sXBP1) and unspliced XBP1 (uXBP1) transcripts were measured by RT-PCR in HEK293 cells transfected with mutant mini-agrin expression vectors. 48 h post-transfection, the proportion of sXBP1 was significantly increased for all the mutant agrins compared to WT (Fig. 6d). Furthermore, RT-qPCR analysis showed that GRP78, GRP94 and CHOP mRNA levels were increased by all the mutant agrins compared to WT agrin (Fig. 6e–g), whereas GADD34 and EDEM1 mRNA levels were only increased in the presence of L2 mutant agrin (Fig. 6h–i). Altogether, these experiments showed that the mutant agrins trigger the activation of the three branches of the UPR pathway.

Misfolded proteins frequently exhibit reduced solubility because of exposed hydrophobic domains. Whole-cell extracts of transfected HEK293 cells were treated with increasing concentrations of detergents at different temperatures (Suppl. Figure 2). While WT agrin was completely soluble, most of L1, L2 and LM agrins remained in the insoluble fraction insoluble even after 15 min heating at 100 °C in the presence of 2% SDS.

### Mutant agrins induce neuronal apoptosis

In case of prolonged or excessive ER stress, UPR triggers apoptosis, also known as ER Stress-Induced Apoptosis. We therefore investigated for a possible neurotoxicity of mutant agrins, which would explain the unusual neurogenic signs observed in the patient. Cell death by apoptosis involves activation of caspase-3 by proteolytic cleavage of its inactive zymogen into activated p17 and p19 fragments.

**Fig. 7:**
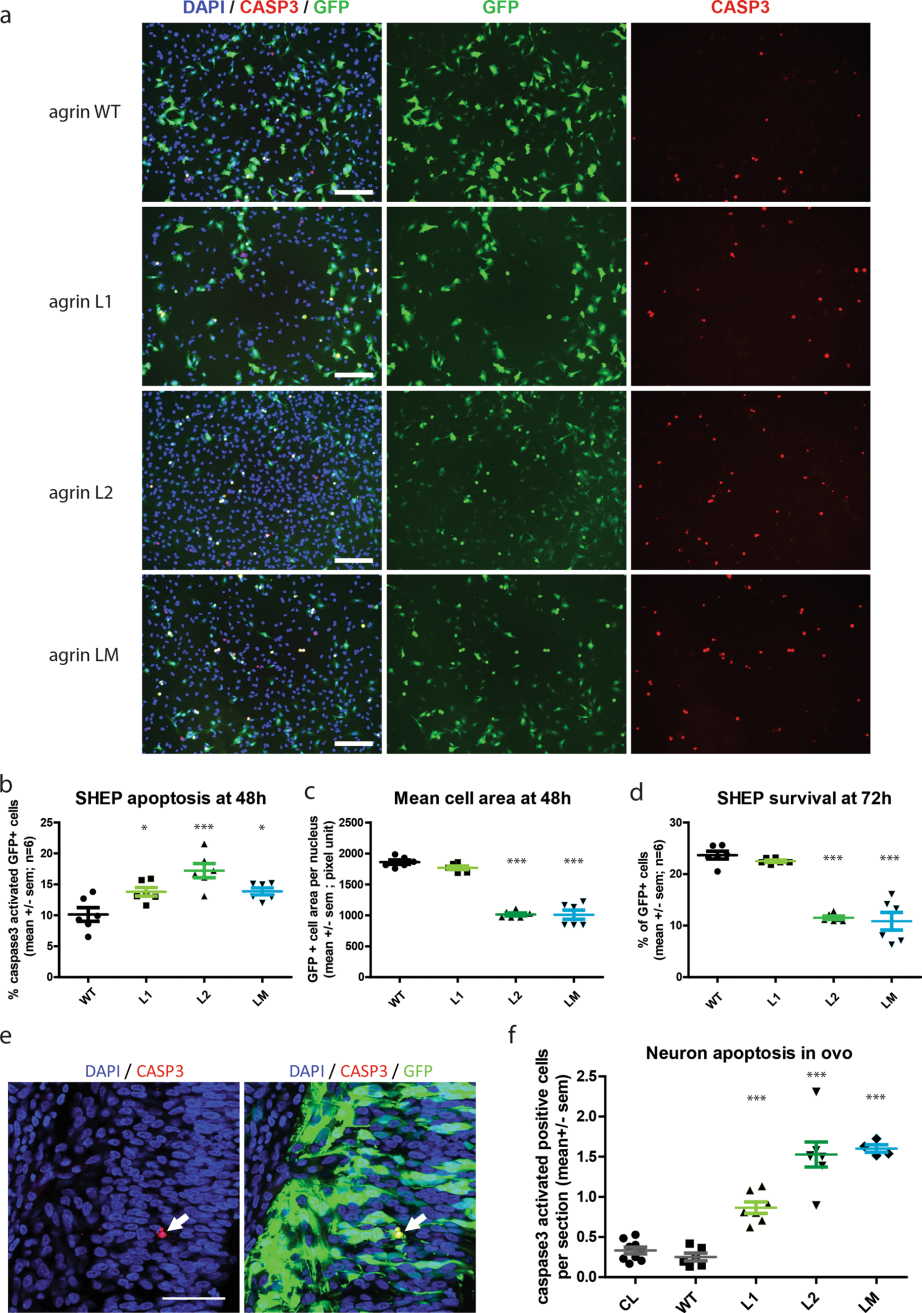
Mutant agrins trigger apoptosis in vitro and *in ovo.* **a** Representative images of SHEP cultures co-expressing mini-agrin and eGFP (green). Blue: nuclei are stained with DAPI. Red: activated caspase-3 immunostaining. Scale bar: 200 µm. **b**–**d** Quantification of activated caspase-3 positive cells and mean cell area 48 h after transfection, and quantification of survival at 72 h (*n* = 3; One-way ANOVA followed by Dunnett's Multiple Comparison Test). **e** Representative confocal images of activated caspase-3 immunostaining (red) in electroporated neurons co-expressing mini-agrin and eGFP (green) *in ovo*. Scale bar: 100 µm. **f** Quantification of activated caspase-3 positive neurons in spinal cord cryosections spanning the entire electroporated spinal cord area (*n* = 6 embryos per condition from 3 independent electroporation sessions; one-way ANOVA followed by Dunnett's multiple comparison test). CL corresponds to the same quantification on the contralateral side of the spinal cord that did not receive the transgene

SHEP cells were transfected with an expression vector for mini-agrin and eGFP. After 48 h, cells were fixed and immunofluorescence for activated caspase-3 was performed (Fig. 7a). Quantification of activated caspase-3-positive cells as well as the average size of transfected cells visualized by eGFP were performed and revealed a significant induction of apoptosis and reduction of the mean cell area (Fig. 7b, c). In a similar experiment, SHEP cells were fixed after 72 h to determine the consequences of mutant agrins expression on survival. The ratio of GFP-positive cells at 72 h and day 0 showed that L2 and LM, but not L1, mutant agrins reduced cell survival (Fig. 7d). Altogether, these results demonstrate that L2 and LM mutations are cytotoxic in vitro.

To confirm these results in vivo, chick neural tubes were electroporated with expression vectors for mutant agrins as in Fig. 3. L1, L2 and LM mutants increased the proportion of cells positive for activated caspase-3 compared either to non-electroporated neurons (CL) or to neurons electroporated with WT agrin (Fig. 7e, f). As expected the L1 mutation was less efficient to trigger caspase 3 activation (Fig. 7f).

Altogether, these results indicated that expression of mutant agrins triggers apoptosis in neurons and that the toxicity of L1 mutant is less severe than L2 and LM mutant.

### When secreted L1, L2 and LM mutant agrins can trigger AChR clustering

**Fig. 8:**
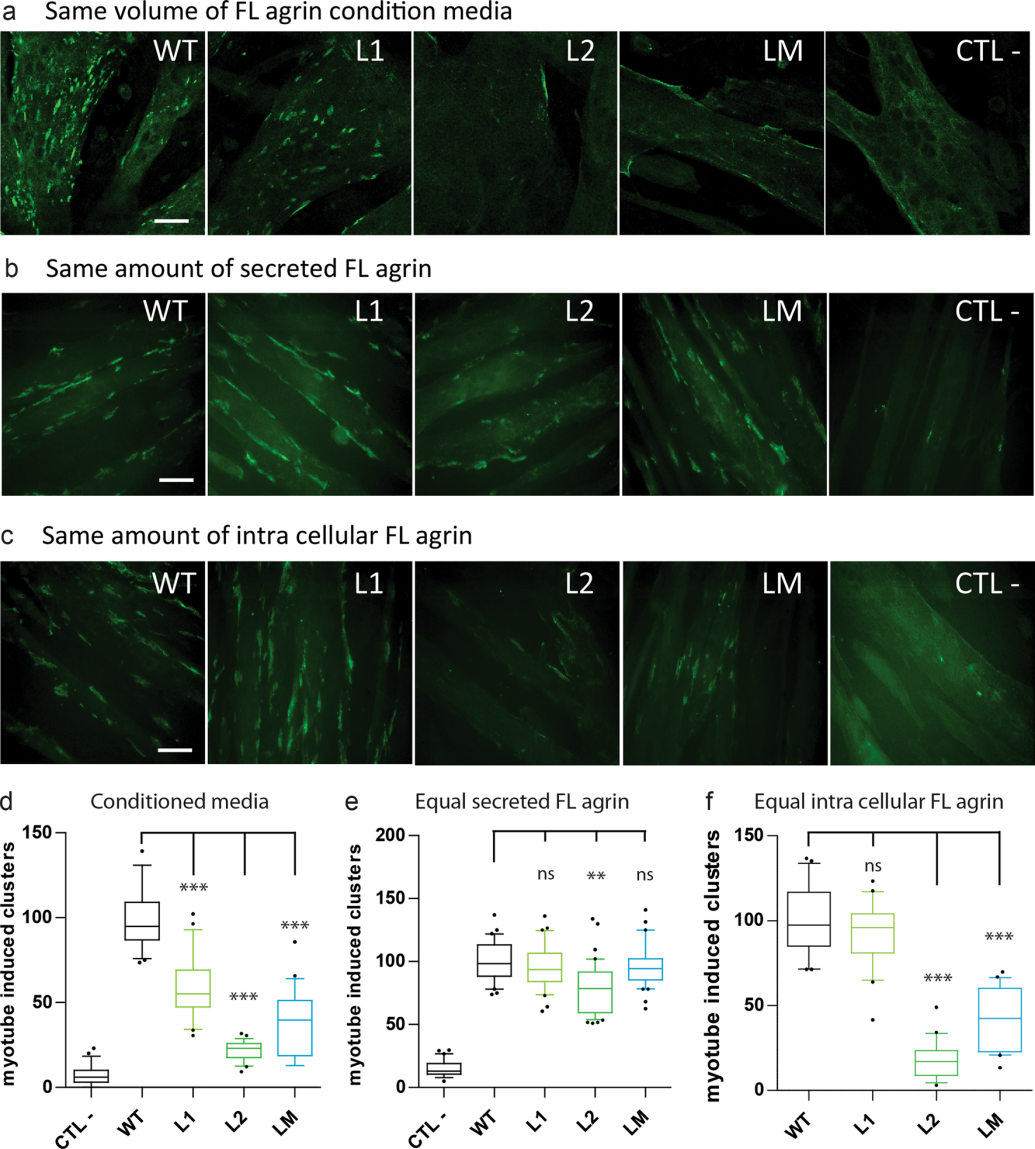
Conditioned media from cells expressing full-length mutant agrins induce less AChR clustering in myotubes. **a**–**c** Pictures of C2C12 myotubes incubated with conditioned media from cells transfected with WT or mutant agrins. Negative control (CTL −) corresponds to a conditioned culture media from cells transfected with an empty vector. AChR clusters were stained with Alexa Fluor 488-conjugated to α-BTX. Scale bar: 50 μm. Myotubes exposed to the same volume of condition media (**a**), or the same amount of secreted agrin (**b**), or the same amount of intracellular agrin from the whole-cell extract resuspended in fresh medium (**c**). Scale bar: 20 µm. **d**–**f** Quantification of the number of AChR clusters per field normalized to the WT agrin condition for the same volume of condition media (**d**), or the same amount of secreted agrin (**e**), or the same amount of intracellular agrin (**f**). Positive control (CTL +) corresponds to C2C12 myotubes treated with 2 nM of purified WT human agrin. Three independent experiments. One-way ANOVA followed by Dunnett's multiple comparison test (***p < 0.001)

To evaluate the consequences of the poor secretion of mutant agrins on the formation of acetylcholine receptor (AChR) clusters, conditioned media was collected from HEK293 cells transfected with expression vectors for full-length WT or mutant agrins. C2C12 myotubes were incubated with an equal volume of each conditioned medium. As expected, the ability of conditioned media from cells expressing mutant agrins induced the formation of significantly less AChR clusters than the medium from WT agrin expressing cells (Fig. 8a, d).

We next investigated whether this loss of AChR-aggregating activity was only due to reduced agrin secretion or if it also involved reduced specific activity of the mutant agrins. For this purpose, the amount of secreted agrin (both WT and mutants) in the culture medium was determined by dot-blot, and C2C12 myotubes were treated with different volumes of conditioned medium, so that each culture plate was provided with the same amount of agrin (Fig. 8b, e). Quantification of the number of AChR clusters indicated that WT, L1 and LM agrins had the same specific activity, suggesting that L1 and LM mutations do not interfere with the intrinsic ability of agrin to trigger AChR clustering. Conversely, the L2 mutant induced slightly less AChR clusters, suggesting that in addition to the secretion defect, the L2 mutation caused a 20% reduction in the AChR clustering activity of agrin (Fig. 8b and 8e).

To investigate if non-secreted agrins that accumulated in the cells were active, whole-cell extracts were prepared from cells transfected with the various agrin constructs. The concentration of agrin in the whole-cell extracts was determined by dot-blot and C2C12 myotubes were treated with similar amounts of intracellular agrin (Fig. 8c). Interestingly, intracellular L1 agrin had the same AChR clustering activity than WT agrin (Fig. 8f). Conversely, intracellular L2 and LM agrins were much less active, suggesting that the proportion of these mutant agins that are not secreted are misfolded and hardly functional.

### iPS-derived motoneurons from Patient 1 secrete less agrin and show reduced survival

**Fig. 9:**
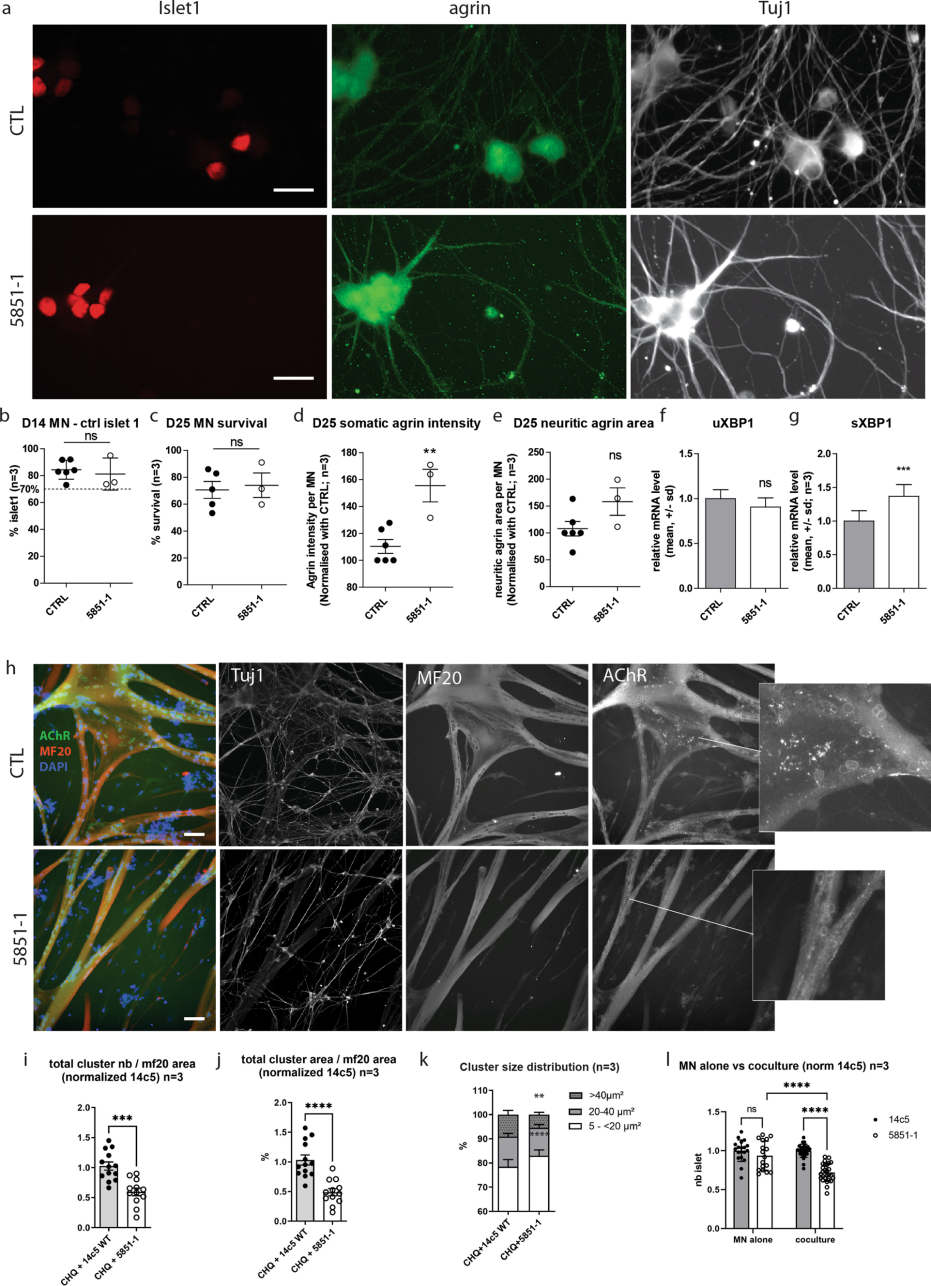
Patient 1 hiPSC-derived motoneurons accumulate mutant agrin, induce less AChR clustering, and show reduced survival rate in co-culture. **a** Representative images of hiPSC-derived MNs after 25 days of differentiation immunostained for Islet1 (red), agrin (green), and Tuj1 (white). Scale bar: 20 µm. **b**, **c** Percentage of surviving hiPSC-derived MNs after 14 and 25 days of differentiation and automatically quantified after immunostaining for Islet1. Data are presented as mean ± SEM, three independent experiments, in quadruplicate and were analyzed by a Student *t* test. **d**, **e** Quantification of the somatic and neuritic intensity and area of agrin staining in hiPSC-derived MNs after 25 days of differentiation. The data were normalized on the number of hiPSC-derived MNs and presented as mean ± SEM, three independent experiments in quadruplicate were analyzed with a Student’s *t* test. **f**, **g** Quantification by RT-qPCR of unspliced (uXBP1) or spliced XBP1 (sXBP1) mRNA level normalized by the total XBP1 (tXBP1) and then by the control MNs at D25. Data are presented as mean ± SD, three independent experiments in triplicate were analyzed by a *t* test (ns *p* > 0.05; ***p < 0.001) **h** Representative images of co-cultures between hiPSC-derived MNs and human primary skeletal muscle cells for 4 days. Co-cultures were characterized by immunostaining for Tuj1, myosin heavy chain (MF20), and AChR. Scale bar: 50 µm. **i**, **j** Quantification of the number and the area of AChR clusters determined by immunostaining with AChR antibody. The number and the area of AChR clusters were normalized on myotube area determined by immunostaining for myosin heavy chain. Data presented as mean ± SEM, three independent experiments in quadruplicate and were analyzed by Student *t* Test. **k** Based on the data generated in panel **h**, and cluster size distribution was determined in function of the area. Data are presented as mean ± SEM, three independent experiments in quadruplicate and were analyzed by Student’s *t* test. **l** Number of hiPSC-derived MNs after 14 days of differentiation and automatically quantified after immunostaining for Islet1. Data are presented as mean ± SEM, three independent experiments, in quadruplicate, and were analyzed by a two-way ANOVA test

To confirm the secretion defect and neurotoxicity in patient’s MNs, hiPSC were generated from Patient 1 and derived into MNs as previously described [29] (Fig. 9a). Differentiation efficiency was monitored by Islet1 quantification in young MNs after 14 days in culture (D14, Fig. 9b). Immunostaining of mature MNs revealed that agrin accumulated in the soma of Patient 1 MNs compared to control hiPSC-derived MNs (Fig. 9d). No significant difference in agrin staining could be detected in the neurites (Fig. 9e). Interestingly, quantification of XBP1 splicing showed a significantly increase compared to control cells, suggesting UPR activation (Fig. 9f-g). Altogether, these experiments indicate that agrin accumulates in the soma of Patient 1 MNs and trigger the UPR.

To investigate if agrin accumulation in the soma correlated with reduced agrin secretion, Patient 1 or control MNs were co-cultured with myotubes (Fig. 9h). AChR clusters were stained with fluorescent α-bungarotoxin. Myotubes co-cultured with Patient 1 MNs produced significantly less (41%) and smaller (51%) AChR clusters than myotubes co-cultured with control MNs (Fig. 9i–k). Altogether, these experiments confirmed that Patient 1-associated mutations lead to the accumulation of agrin within the soma and, consequently, have a reduced capacity to trigger the formation of AChR clusters.

To confirm neurotoxicity, neuronal viability was also investigated. Patient and control mono-cultures of MNs had the same survival values (Fig. 9c). Conversely, when co-cultured with myotubes, the survival of Patient 1 MNs was significantly reduced (28%) compared to control MNs (Fig. 9l).

## Discussion

Three patients with initial suspicion of early onset motoneuron disorder (SMA) were diagnosed with a neuromuscular transmission defect and shown to carry mutations in the LG2 domain of agrin causing defects in agrin secretion by MNs. These patients provide evidence for a new pathomechanism in CMS whereby protein misfolding in MNs causes a deficit in agrin secretion by MNs and SMA-like features.

Most *AGRN* mutations identified in CMS patients so far are located in the LG2 domain [18, 23, 27, 35, 47, 50]. The first CMS patient with a mutation in the *AGRN* gene carried an homozygous G1709R mutation that did not impair agrin production. This mutation did not prevent MuSK activation by agrin and did not affect agrin-induced AChR aggregation in cultured myotubes. However, neuromuscular junctions were disorganized in the patient and in mouse muscles electroporated with the mutant agrin [18]. Conversely, the heteroallelic V1727F mutation was shown to decrease agrin-induced phosphorylation of MuSK and AChR clustering [27]. This mutation was subsequently shown to impair neural agrin secretion in heterologous cells and molecular modelisation predicted that the V1727F mutation primarily disrupted the A/y splice insert of neural agrin and thus altered its interaction with heparin and LRP4 [42]. Interestingly, this mutation that caused misfolding of agrin increased interaction with dystroglycan [27]. It would be interesting to evaluate if the L1 mutation (R1671Q) also displays increased interaction with dystroglycan. This would explain the reduced diffusibility we observed for this mutant.

The new mutations presented here further increase the diversity of pathophysiological mechanisms associated with CMS caused by mutations in *AGRN*, emphasizing the crucial role of the LG2 domain. L1 and L2 mutations that affect arginines 1671 and 1698 are localized in loop regions of the LG2 beta sheets, and both presumably form H bonds that stabilize the folding of the protein. Their respective substitution by a glutamine and a proline would abrogate these bonds and therefore favor misfolding. Similarly, for the LM mutation, the substitution of a leucine by a proline at position 1664 is susceptible to cause misfolding of the LG2 domain. The fact that mutations in an LG domain of agrin can cause protein misfolding is also consistent with the observation that a missense mutation in the G domain of laminin-5 causing junctional epidermolysis bullosa affects proper folding of the protein and results in the retention of the mutant polypeptide within the ER [43]. Of note, an R1698C substitution was previously described in a heteroallelic patient at the same position as our L2 variant (L1698P) and was reported to cause instability of agrin [47]. This illustrates that substitutions by different amino acids at a given position can differentially affect a protein, and therefore can possibly induce different clinical manifestations.

Misfolding frequently causes the formation of protein aggregates, as in Amyotrophic Lateral Sclerosis caused by TDP43 mutations, or in neurological diseases caused by mutations in Tau or α-synuclein [5, 22, 37]. As often observed with misfolded proteins, the L2 and LM mutations cause retention in the ER lumen. Conversely, although less secreted than WT agrin, the L1 mutant is processed all along the secretion pathway and is secreted to form poorly diffusible extracellular aggregates. Such accumulation of extracellular protein aggregates is reminiscent of amyloid aggregates in the brain of patients with Alzheimer’s disease [39]. Interestingly, agrin was shown to co-localize with fibrillar amyloid-beta (Aβ) deposits in senile plaques [6]. Similarly in Parkinson’s disease, agrin was shown to associate with α-synuclein in Lewy bodies of the substantia nigra [26].

Our results indicate that in the presence of patients with a clinical presentation of SMA but without mutation in the *SMN1* gene, it can be worth to look for mutations in *AGRN*. Aggregates of misfolded proteins are often associated with toxicity due to ER stress and increased UPR. It is therefore conceivable that agrin aggregates interfere with normal MNs function. This is consistent with the fact that expression of these mutant agrins in vitro in SHEP or in vivo in chicken MNs induces the activation of the pro-apoptotic caspase 3. WT agrin overexpression had no detectable effect on MNs viability, but we nevertheless evaluated the viability of Patient 1 hiPS-derived MNs to eliminate a possible bias due to the overexpression of mutant proteins. As expected, when co-cultured with muscle cells, the hiPS-derived MNs carrying L1 and L2 mutations exhibited reduced viability compared to controls. Viability was specifically impaired in co-cultures and not in MNs mono-cultures. Muscle cells have trophic effects on MNs and reduced viability in co-cultures probably reflects the fact that the defect in NMJ formation exacerbates the difference in viability with control MNs. Alternatively, the different culture media used for mono and co-cultures could also contribute to this difference. The finding of a significant increase in XBP1 splicing in Patient 1 hiPSC-derived MNs after 25 days of culture indicates an activation of the UPR. The fact that this is not associated with increased cell death could mean that UPR is not responsible for neuronal toxicity of mutant agrins. Alternatively, since ER stress affects viability progressively, it is also possible that cell death would occur at later stages of culture. In this respect, it is worth noticing that to observe cellular stress in hiPSC-derived MNs, it usually takes culture times longer than 25 days. Such UPR activation is rarely observed in hiPSC-derived MNs after only 25 days of culture as recently described in SMA hiPSC-derived MNs [8]. Finally, reduced MN survival in co-cultures and not in mono-cultures could be indicative of non-cell-autonomous mechanisms. Indeed, the pathophysiology of motoneuron diseases have been shown to involve both cell-autonomous and non-cell-autonomous mechanisms [2, 3]. The non-cell-autonomous effects have been described in vitro to be either toxic [32] or protective [19].

Altogether, ER stress, induction of UPR, and caspase activation in MNs expressing the mutant agrins are consistent with the fact that the clinical presentation of the three patients was evocative of SMA. SMA features are probably not due to the deficit in agrin secretion per-se, since CMS patients with Myo9A mutations have a deficit in agrin secretion, but, although severely affected, do not present SMA features [34]. Our results show for the first time that *AGRN* mutations can induce SMA features. Whether this is due to the toxicity of neuronal aggregates of mutant agrin or to a yet undefined role of agrin in MNs remains to be determined. The fact that agrin inactivation in mouse does not seem to perturb MNs before NMJ formation [25] pleads in favor of a toxicity of agrin aggregates. However, a role of agrin in adult MNs cannot be excluded.

## Supplementary Information

Below is the link to the electronic supplementary material.

Suppl. Figure 1 Rabbit anti-human agrin antibody specificity. a. Confocal images of a primary culture of mouse MNs. Cells expressing human agrin coexpress eGFP (green). As expected, the rabbit antibody against human agrin detects a signal (red) only in transfected cells. b. Neither the rabbit pre-immune serum (pre-sera), nor the rabbit serum after immunization against agrin, stains mouse NMJ. As a positive control, agrin was visualized on mouse muscle sections with another primary antibody (dilution 1:250; green) [12]. α-bungarotoxin staining of AChR on NMJs is shown in red and DAPI-stained nuclei are shown in blue. The white arrow indicates the location of the agrin at the NMJ. Scale bar: 25 μm (TIF 9100 kb)

Suppl. Figure 2 L1 mutant extracellular aggregates. a. Confocal images of agrin immunostaining (red) on non-permeabilized mouse MN primary cultures transfected with WT or L1 mini-agrin IRES GFP (green) constructs immunostaining conditions. Six left panels: Scale bar: 20 µm. Higher magnification (2 right panels): Scale bar: 5 µm. b. Quantification of the agrin staining dispersion in function of the distance to the middle of the axon. Each dot corresponds to 0.06 µm steps. Line graph representing the agrin concentration (mean of the total intensity, % +/- sem) on at least 18 MN axons per condition. c. Mini-agrin western blot after differential solubilization in 1% triton, 2% SDS (SDS soluble). SDS insoluble corresponds to the insoluble protein pellet that remains after heating and sonication in 2% SDS. (TIF 3652 kb)

Suppl. Figure 3 : Confocal images of spinal cord cryosection from chick neural tube electroporated with mutant mini-agrins IRES eGFP constructs. Electroporated neurons are identified by eGFP and MNs are identified by Islet1/2 immunostaining. Scale bar: 200 µm (TIF 4171 kb)
