# Supplementary figures and images for: Severe congenital myasthenic syndromes caused by agrin mutations affecting secretion by motoneurons
Source: Acta Neuropathol. 2022 Aug 10;144(4):707–31. doi: 10.1007/s00401-022-02475-8 (PMC9468088; doi:10.1007/s00401-022-02475-8)

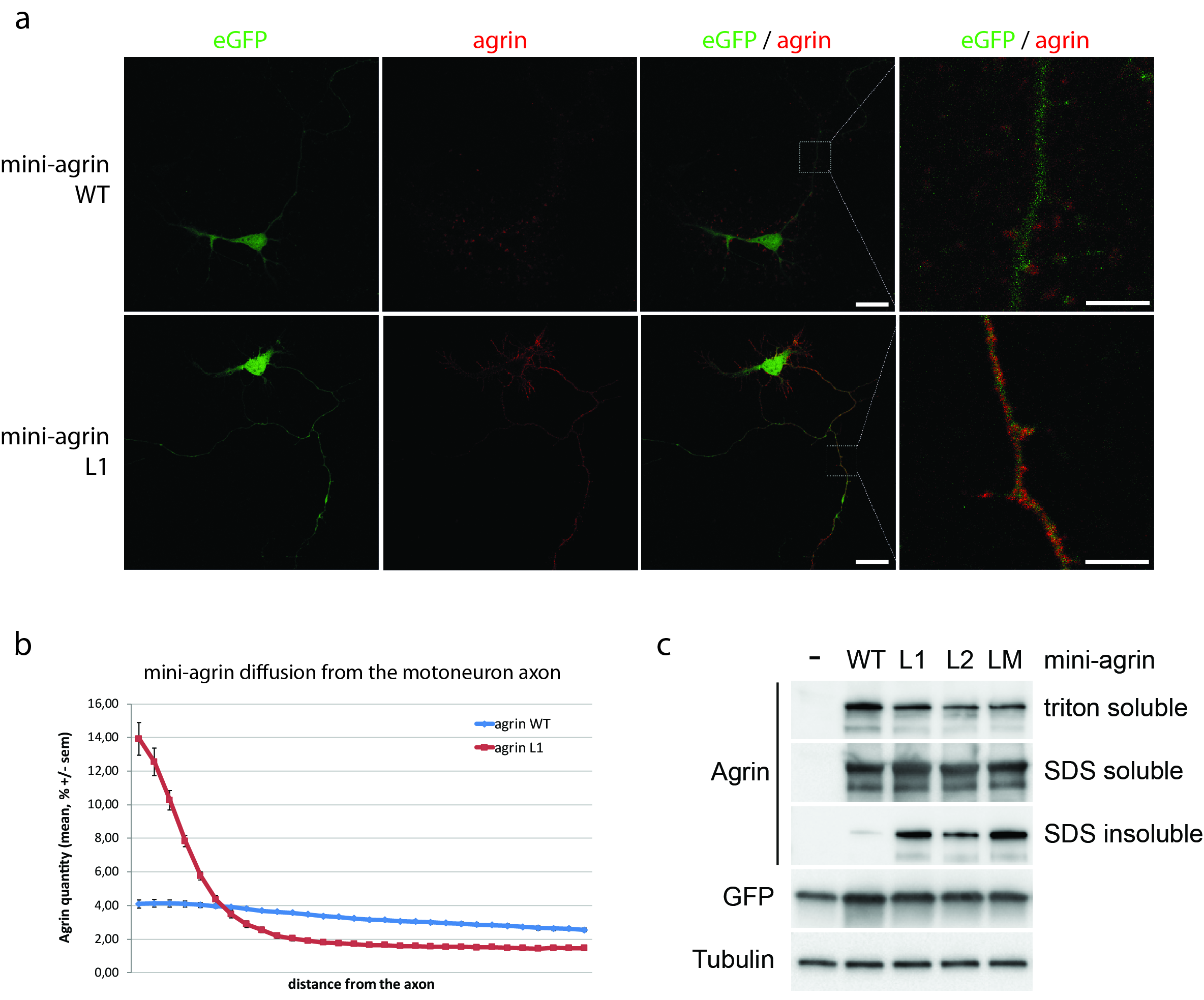

Supplement: Supplementary file 1 — Suppl. Figure 1 Rabbit anti-human agrin antibody specificity. a. Confocal images of a primary culture of mouse MNs. Cells expressing human agrin coexpress eGFP (green). As expected, the rabbit antibody against human agrin detects a signal (red) only in transfected cells. b. Neither the rabbit pre-immune serum (pre-sera), nor the rabbit serum after immunization against agrin, stains mouse NMJ. As a positive control, agrin was visualized on mouse muscle sections with another primary antibody (dilution 1:250; green) [12]. α-bungarotoxin staining of AChR on NMJs is shown in red and DAPI-stained nuclei are shown in blue. The white arrow indicates the location of the agrin at the NMJ. Scale bar: 25 μm (TIF 9100 kb) [file 401_2022_2475_MOESM1_ESM.tif]

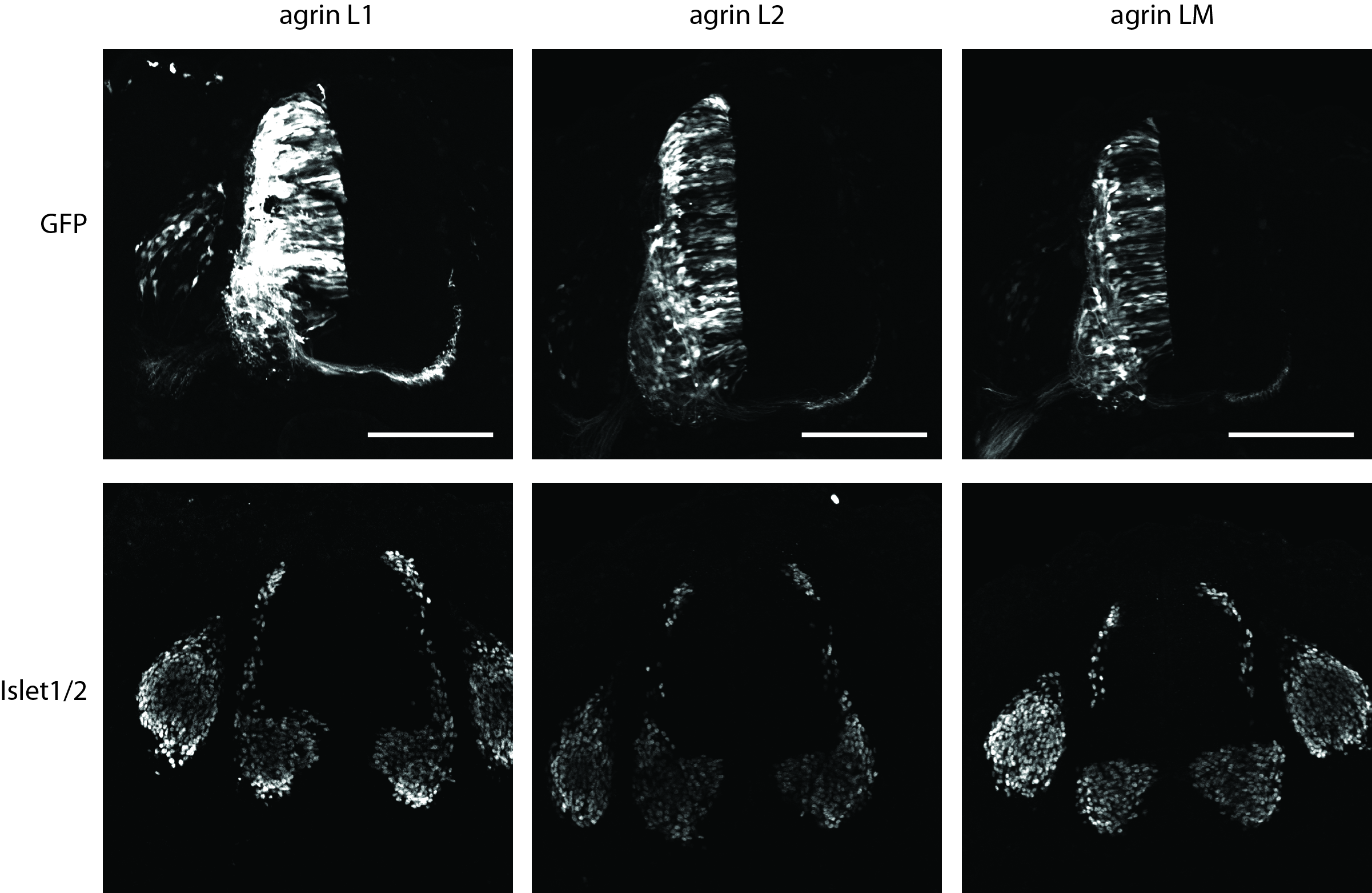

Supplement: Supplementary file 2 — Suppl. Figure 2 L1 mutant extracellular aggregates. a. Confocal images of agrin immunostaining (red) on non-permeabilized mouse MN primary cultures transfected with WT or L1 mini-agrin IRES GFP (green) constructs immunostaining conditions. Six left panels: Scale bar: 20 µm. Higher magnification (2 right panels): Scale bar: 5 µm. b. Quantification of the agrin staining dispersion in function of the distance to the middle of the axon. Each dot corresponds to 0.06 µm steps. Line graph representing the agrin concentration (mean of the total intensity, % +/- sem) on at least 18 MN axons per condition. c. Mini-agrin western blot after differential solubilization in 1% triton, 2% SDS (SDS soluble). SDS insoluble corresponds to the insoluble protein pellet that remains after heating and sonication in 2% SDS. (TIF 3652 kb) [file 401_2022_2475_MOESM2_ESM.tif]

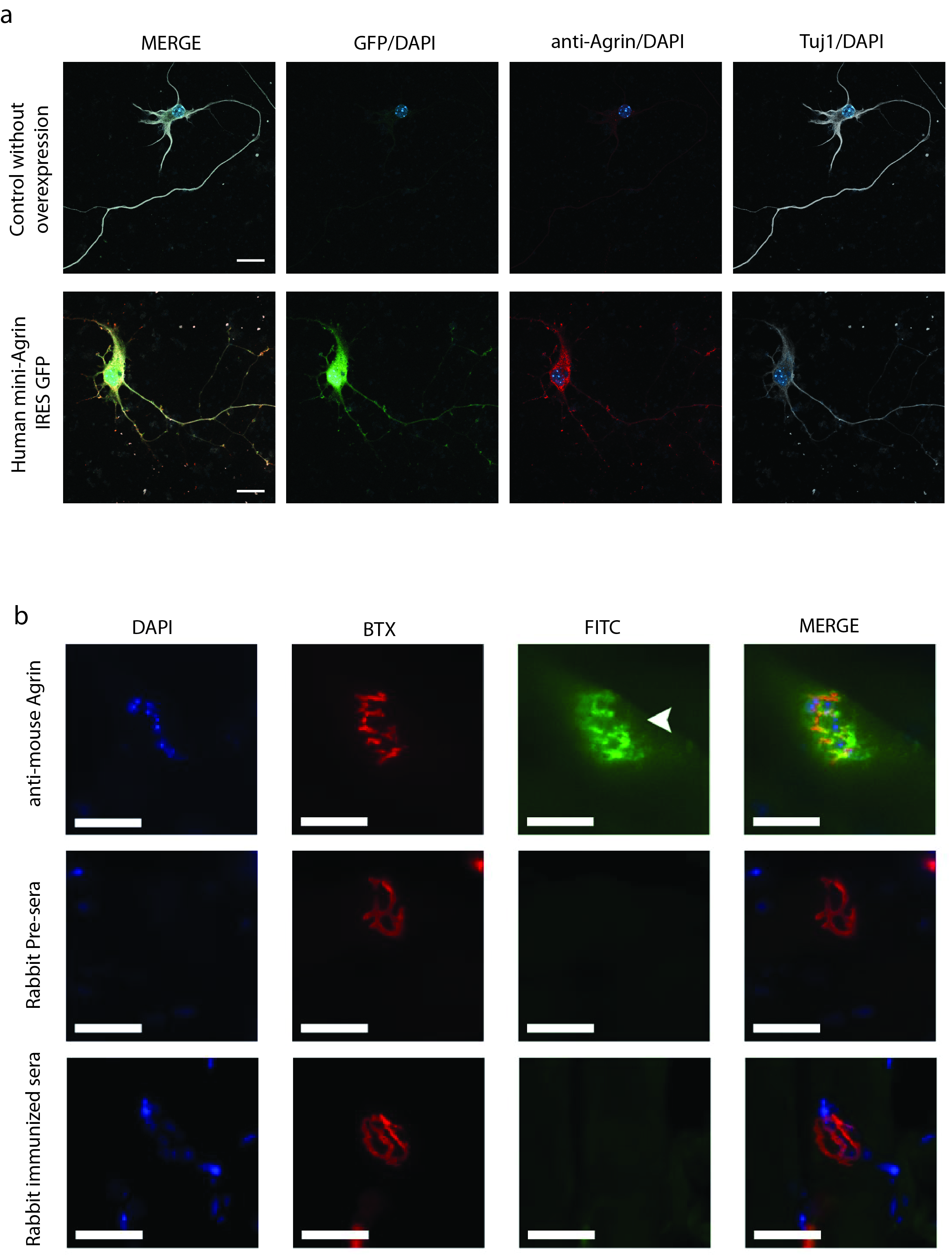

Supplement: Supplementary file 3 — Suppl. Figure 3 : Confocal images of spinal cord cryosection from chick neural tube electroporated with mutant mini-agrins IRES eGFP constructs. Electroporated neurons are identified by eGFP and MNs are identified by Islet1/2 immunostaining. Scale bar: 200 µm (TIF 4171 kb) [file 401_2022_2475_MOESM3_ESM.tif]
